# AI-Driven Energy-Efficient Routing in IoT-Based Wireless Sensor Networks: A Comprehensive Review

**DOI:** 10.3390/s25247408

**Published:** 2025-12-05

**Authors:** Sumendra Thakur, Nurul I. Sarkar, Sira Yongchareon

**Affiliations:** Computer and Information Sciences, Auckland University of Technology, Auckland 1010, New Zealand; sumendra.thakur@autuni.ac.nz (S.T.); sira.yongchareon@aut.ac.nz (S.Y.)

**Keywords:** internet of things, wireless sensor networks, energy efficiency, routing optimization, artificial intelligence (AI), machine learning (ML)

## Abstract

Efficient routing remains the linchpin for achieving sustainable performance in Wireless Sensor Networks (WSNs) within the Internet of Things (IoT). However, traditional routing mechanisms increasingly struggle to cope with the growing complexity of network architectures, frequent changes in topology, and the dynamic behavior of mobile nodes. These issues contribute to data congestion, uneven energy consumption, and potential communication breakdowns, underscoring the urgency for optimized routing strategies. In this paper, we present a comprehensive review of over 100 studies of spanning conventional and AI-enhanced energy-efficient routing techniques. It covers diverse approaches, including metaheuristics, machine learning, reinforcement learning, and AI-based cross-layer methods aimed at improving the performance of WSN-IoT systems. The key limitations of existing solutions are discussed along with performance metrics such as scalability, energy efficiency, throughput, and packet delivery. We also highlight various research challenges and provide research directions for future exploration. By synthesizing current trends and gaps, we provide researchers and practitioners with a structured foundation for advancing intelligent, energy-conscious routing in next-generation IoT-enabled WSNs.

## 1. Introduction

Efficient and adaptive routing is central to the long-term sustainability of IoT-enabled Wireless Sensor Networks (WSNs) [1,2]. Wireless Sensor Networks (WSNs) offer numerous advantages that make them attractive for a wide range of IoT applications. First, they are financially affordable: many WSN nodes are low-cost and require minimal infrastructure, making deployment feasible even in remote or wide-area environments [3]. Second, WSNs provide real-time monitoring and automated data collection, allowing prompt responses to environmental changes or system events. The infrastructure-less, wireless architecture enables rapid installation and flexibility, particularly where laying cables is impractical. The compatibility with a wide set of wireless protocols (such as LoRa, ZigBee, Bluetooth, Wi-Fi, and LPWAN) allows WSNs to integrate easily with diverse systems and platforms. The low maintenance requirements and long battery life further enhance their suitability for unattended or hard-to-access locations. Thanks to these advantages, WSNs are widely used in industrial automation, environmental monitoring, smart agriculture, smart homes, energy management, and many other fields [4,5]. The significance of routing lies in its dual impact: it not only determines communication reliability but also governs the energy consumption of battery-powered sensor nodes, thereby shaping the overall lifetime of the network [6]. Yet, routing in WSNs presents formidable challenges. Sensor nodes are often resource-constrained and deployed in dynamic or inaccessible environments, where frequent maintenance is impractical [7]. High node density, uneven traffic distribution, and mobile topologies exacerbate congestion and accelerate energy depletion [8]. Moreover, routing is inherently computationally complex commonly modelled as an NP-hard problem which makes difficult to balance competing objectives such as energy efficiency, scalability, throughput, latency, and quality of service (QoS) [9]. Conventional protocols, which were largely designed to minimize hop count or maximize throughput, struggle under these conditions, leading to premature node failures, network partitioning, and reduced reliability [10]. To address these limitations, researchers have increasingly turned to optimization-driven and intelligent methodologies. Artificial Intelligence (AI) techniques—including metaheuristics, machine learning, reinforcement learning, and deep learning—offer capabilities for real-time adaptation, predictive decision-making, and self-optimization [11,12]. These approaches offer promising alternatives to static rule-based methods, enabling networks to adapt to environmental changes and dynamic heterogeneous traffic demands.

However, despite rapid progress, the existing body of survey literature on WSN routing remains fragmented. Early reviews primarily focused on classical clustering and hierarchical methods, offering valuable but narrow insights into energy-aware design [13]. More recent surveys have incorporated AI-driven strategies, but typically with limited scope, for example, focusing only on machine learning algorithms or application-specific scenarios. In contrast to earlier reviews, this paper provides a unified taxonomy that integrates both classical routing protocols and AI-enhanced approaches, spanning cross-layer design, metaheuristics, and deep learning frameworks.

The rationale for this survey is therefore twofold. First, it addresses the absence of a consolidated review that bridges traditional and AI-driven perspectives, highlighting their complementary strengths and persistent limitations. Second, it identifies underexplored areas—such as hybrid AI models, lightweight reinforcement learning for constrained devices, and federated learning architectures—that hold significant potential for advancing next-generation IoT-WSNs. By systematically synthesizing these dimensions, this survey offers both a critical reference point and a forward-looking roadmap for researchers and practitioners aiming to design scalable, intelligent, and energy-efficient routing frameworks.

### 1.1. Research Background and Motivation

The motivation for this review paper stems from the urgent need to address persistent challenges of energy efficiency, scalability, and reliability in routing protocols for IoT-enabled wireless sensor networks (WSNs), which support critical applications in smart cities, environmental monitoring, agriculture, industry, and healthcare. As IoT deployments become larger, more mobile, and more heterogeneous, conventional routing protocols including cross-layer and cluster-based schemes which struggle to adapt routes in real time, balance traffic, and prevent rapid energy depletion under dynamic conditions, leading to congestion, hotspots, and premature node failures.

Artificial intelligence (AI)–driven routing has emerged as a promising response to these limitations because learning- and optimization-based methods can continuously adjust routing decisions to current network state instead of relying on fixed heuristics. By using machine learning, reinforcement learning, and metaheuristic optimization, AI-based protocols can, for example, predict traffic patterns, select energy-balanced cluster heads, and choose routes that jointly optimize residual energy, link quality, delay, and packet delivery ratio, thereby extending network lifetime and improving quality of service compared with traditional algorithms. At the same time, deploying AI in WSNs is non-trivial due to severe resource constraints on sensor nodes; therefore, most AI-related computation and model training are offloaded to more capable gateways, cluster heads, and fog/edge layers, while sensor nodes execute only lightweight inference to keep local overhead low. Therefore, there is a critical and timely need for a comprehensive, up-to-date review to evaluate current progress, systematically compare state-of-the-art approaches, identify persistent research gaps, and inspire the development of innovative, sustainable routing solutions that can guarantee the adaptability and resilience of future IoT-WSN infrastructures in a rapidly evolving technological landscape.

### 1.2. Research Challenges and Questions

Despite substantial progress in optimizing routing for IoT-based WSNs, several challenges persist in realizing truly intelligent and energy-aware communication systems. One of the foremost difficulties lies in designing route optimization schemes that can perform efficiently under resource constraints and varying deployment environments. The existing protocols often struggle to maintain a balance among energy consumption, latency, and throughput when operating across dense and large-scale IoT infrastructures [9]. Moreover, heterogeneous node capabilities, fluctuating traffic patterns, and environmental interference further complicate route discovery and maintenance. Developing universally adaptive models that can accommodate these dynamics without excessive computational, or communication overhead remains a major obstacle.

The integration of artificial intelligence into routing introduces both opportunities and new complications. Although machine learning, reinforcement learning, and metaheuristic algorithms have shown remarkable potential in enhancing decision-making and extending network lifetime [14], they also raise concerns about scalability and real-time adaptability. Training AI models within low-power sensor environments is challenging due to limited processing capacity and storage. Furthermore, the lack of standardized datasets and evaluation benchmarks makes it difficult to compare AI-based routing frameworks objectively with traditional counterparts. Achieving efficient on-device learning, minimizing training latency, and reducing the communication costs associated with model updates remain open areas of investigation.

Looking ahead, emerging research must also address the broader issues shaping the future of WSN-IoT routing. With the increasing adoption of mobile sinks, drone-assisted communication, and hybrid static–dynamic network topologies, maintaining energy balance and reliability in real-time remains difficult. Security and fault tolerance [15] are equally critical, as attacks on routing layers can disrupt essential IoT services in industrial and healthcare domains. Novel directions such as bio-inspired optimization and lightweight cross-layer architectures show promise, yet their practical implementation requires careful calibration among intelligence, energy efficiency, and network resilience. Bridging these gaps is essential for developing next-generation routing protocols capable of autonomous adaptation and long-term sustainability in complex IoT ecosystems.

A central design challenge in wireless sensor networks (WSNs) is overcoming severe energy limitations, as sensor nodes are usually battery-powered and often cannot be replaced or recharged after deployment. To extend network lifetime, routing protocols are specifically engineered to minimize energy consumption during data transfer and network operation. This is achieved through several ways: employing multi-hop communication to avoid costly long-range transmissions, organizing nodes into energy-balanced clusters with rotating cluster heads, adaptively adjusting transmission power based on distance, and selecting data paths according to residual node energy and network conditions. Many recent protocols also use hybrid metaheuristic or learning-based techniques to optimize cluster head selection and routing based on energy efficiency criteria. As a result, nearly all modern WSN routing solutions place energy conservation at the core of their protocol design and performance evaluation, reflecting the unique resource constraints of these networks.

This survey addresses the following core research questions (RQs) that are pivotal for progressing toward more intelligent and energy-conscious routing in IoT-integrated Wireless Sensor Networks (WSNs):

RQ1. What are the current state-of-the-art route optimization techniques used in IoT-based WSNs?
RQ2. What thorough evaluation and analysis can be conducted to investigate classical and AI-based energy-efficient routing optimization in WSN-IoT networks?
RQ3. What are the emerging trends, open research challenges, and future directions in developing energy-aware routing protocols for next-generation WSN-IoT systems?

These above questions are designed to offer a structured exploration of both the current capabilities and the untapped potential in the domain of routing optimization. The first question (RQ1) focuses on capturing a comprehensive overview of state-of-the-art techniques, particularly those tailored for resource-constrained and large-scale IoT deployments. The second (RQ2) aims to compare conventional algorithmic models and AI-driven methods, including machine learning, reinforcement learning, and metaheuristics, by examining their suitability in dynamic and heterogeneous network scenarios. The final question (RQ3) aims to identify prospective developments and innovative strategies that may shape the future of WSN routing, including bio-inspired algorithms, federated learning, and lightweight cross-layer designs. These questions establish a framework for understanding the technological landscape and the forward trajectory of research in this field.

### 1.3. Research Scope and Contributions

To explore the above questions, this review synthesizes insights from over 100 peer-reviewed journals and conference publications sourced from respected academic databases, including IEEE Xplore, ACM Digital Library, Elsevier, MDPI, and ScienceDirect. The literature reviewed was selected based on relevance to a focused set of search terms related to energy-efficient routing, AI in WSNs, IoT-based optimization, and intelligent communication protocols.

The main contributions of this paper are summarized as follows:

- Comprehensive Literature Survey: We conduct a broad review and categorization of existing energy-efficient routing techniques, distinguishing between conventional methods and AI-enhanced strategies, including machine learning, metaheuristic algorithms, and cross-layer optimization approaches. The surveyed protocols are systematically classified based on key performance attributes such as energy consumption, scalability, packet delivery ratio, latency, and protocol adaptability to IoT-specific constraints.
- Comparative Analysis: We present a detailed evaluation of the strengths, weaknesses, and application scenarios for traditional and AI-driven routing techniques, highlighting each technique’s practical benefits and limitations.
- Research Gap/Challenges analysis led to future directions: Our analysis reveals several unresolved challenges, such as inefficient cluster head selection, energy bottlenecks in multi-hop routing, and limited real-time adaptability of routing algorithms in heterogeneous IoT environments. The paper recommends promising avenues for further exploration, including the development of energy-aware cluster formation mechanisms, lightweight and secure routing protocols for constrained devices, and the integration of AI models capable of self-learning and online optimization in dynamic WSN topologies.

Through these contributions, the paper aims to serve as both a reference point and a roadmap for researchers, developers, and system designers working on intelligent routing frameworks in IoT-driven wireless sensor networks.

### 1.4. Structure of This Paper

**Figure 1:**
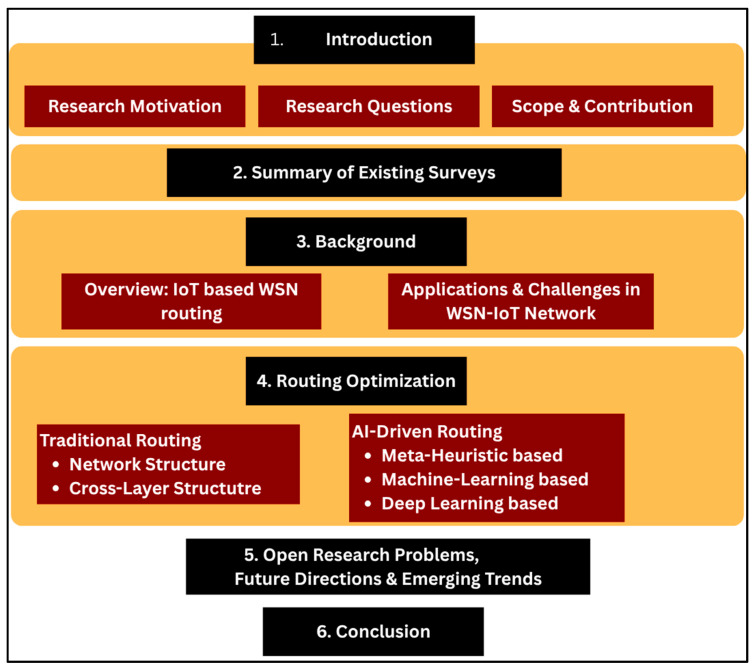
Organization and structure of the paper.

The organization and structure of the paper are shown in Figure 1. In Section 2, we provide a summary of existing papers. In Section 3, we briefly describe the WSN applications and challenges faced in this network. In Section 4, we conduct our main discussion and literature review on transitioning from traditional-based routing to AI-driven routing in the WSN-IoT network. We present the need for routing optimization in WSN-IoT networks, followed by a discussion of different approaches to route optimization. Section 5 discusses future directions and research opportunities. Finally, Section 6 presents the conclusion of this review paper.

## 2. Summary of Existing Surveys

Over the past few years, numerous survey papers have explored energy-efficient routing strategies in Wireless Sensor Networks (WSNs), particularly as they intersect with IoT applications. These reviews have contributed significantly to understanding routing challenges, performance trade-offs, and the evolution of protocol designs. Table 1 presents a comparative summary of key existing survey works across parameters such as clustering techniques, AI-based optimization strategies, application focus, and route optimization techniques such as Network Structure (NS), Cross-Layer (CL), Meta-Heuristics (MH), Machine Learning (ML), and Deep Learning (DL).

Several earlier surveys, such as those by Aghbari et al. [16] and Agarkar et al. [17], concentrated on traditional routing protocols, emphasizing hierarchical models, energy conservation, and routing trade-offs. While these works provide foundational insights into classical optimization techniques, they typically do not incorporate AI-driven methods, which are increasingly relevant for adaptive and intelligent routing.

Recent contributions, such as Poornima et al. [18] and Priyadarshi [14], began integrating machine learning (ML) and deep learning (DL) perspectives, particularly for routing in dynamic and heterogeneous IoT environments. However, these reviews tend to focus narrowly on specific AI categories, such as ML-based algorithms, without presenting a comprehensive taxonomy that includes metaheuristic or cross-layer strategies.

A few surveys, such as Ramya & Brindha [19] and Singh et al. [20], explored application-specific implementations, including cluster head selection and agricultural monitoring, which provided deeper insight into targeted domains but lacked a broader comparative analysis across architectural layers and routing paradigms. Additionally, works such as Nag et al. [21] addressed security and coverage challenges in routing, reflecting the multidisciplinary nature of modern WSN issues, yet did not explicitly focus on energy-efficient optimization frameworks.

Notably, cross-layer designs and emerging hybrid routing strategies remain underexplored in existing reviews. For instance, Martalò et al. [22] focused on secure cross-layer routing but did not examine AI-enhanced techniques. Similarly, while some studies address multi-hop routing and load balancing, e.g., Sahu & Veenadhari [23], they often fail to provide a deeper exploration of scalability, learning adaptability, or protocol-level evolution.

Given these gaps, the present survey aims to provide a comprehensive and detailed review that unifies classical and AI-driven approaches within a common framework. It provides a comparative taxonomy that includes Network-structure-based and cross-layer routing (traditional), as well as Metaheuristics, machine learning, and deep learning techniques (AI-driven). Moreover, this review identifies existing limitations in current methodologies and discusses research opportunities for developing more adaptive, scalable, and intelligent routing frameworks suited to next-generation IoT-WSN systems.

**Figure 2:**
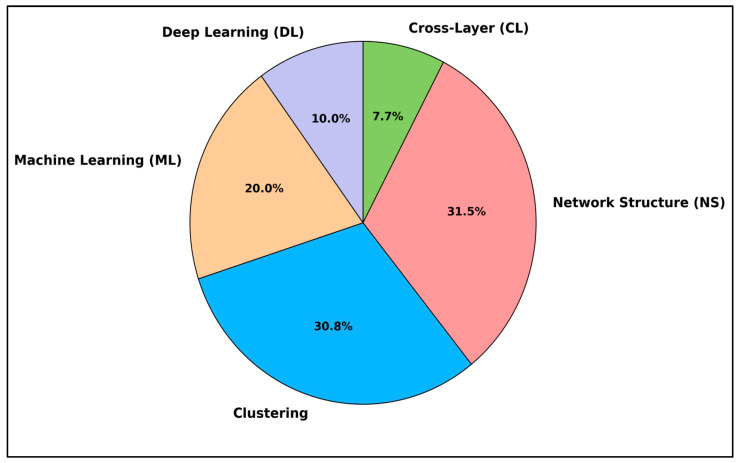
Focus areas discussed in this paper.

Figure 2 summarises the focus areas found across the survey papers reviewed in this study. Most existing work focuses on application-driven contexts, particularly within the WSN and IoT domains, where energy-efficient routing is crucial for achieving real-world performance and sustainability. Classical approaches, particularly those based on network structure, continue to be widely discussed, demonstrating their enduring relevance in WSN routing research. Clustering techniques also appear frequently, likely due to their impact on reducing communication overhead and improving network longevity. However, as the chart shows, machine learning and deep learning approaches, while increasingly popular—are still relatively underexplored in the literature. Similarly, cross-layer optimisation offers a more integrated view of communication efficiency and has received limited attention.

These observations underscore the importance of this survey, which seeks to bridge these gaps by integrating classical methods with emerging AI-based routing strategies and pinpointing areas that require further investigation.

## 3. IoT-Based Wireless Sensor Networks

### 3.1. Background and Overview

**Figure 3:**
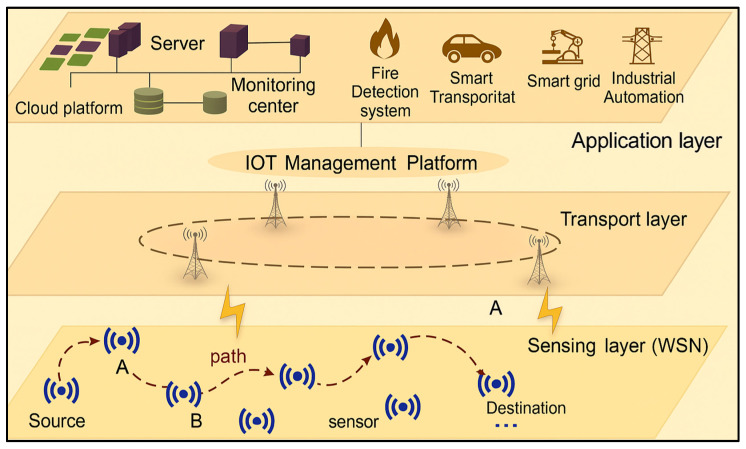
Typical architecture of an integration between WSN-IoT Network [47].

The Internet of Things (IoT) provides a framework that enables physical devices, sensors, and systems to communicate, share data, and interact with each other over the internet [36,37]. When this innovative paradigm intersects with the domain of Wireless Sensor Networks (WSNs), it significantly amplifies the capabilities of both technologies [38]. A WSN, comprising a network of strategically deployed sensors and routing nodes, enhances IoT functionalities by enabling continuous, real-time data acquisition and analysis across diverse environments [39]. The integration of WSNs into IoT infrastructures not only improves system responsiveness but also strengthens predictive capabilities, allowing for proactive decision-making and more efficient resource utilization [40]. As illustrated in Figure 3, the routing process in WSN-IoT network, where sensor nodes, such as accelerometers, pressure sensors, and temperature sensors, first capture environmental data and forward it using wireless communication protocols to a local gateway [41,42].

Moreover, routing protocols are needed in a WSN depend largely on the network’s physical size and node placement. For small areas, typically less than about 10,000 square meters (100 m × 100 m) where nodes are close enough to the gateway, direct transmission is usually practical and energy efficient. However, in larger networks stretching over thousands of square meters, direct communication becomes costly in terms of energy, especially for far-away nodes. In these cases, routing protocols are essential to enable multi-hop paths, allowing intermediate nodes to relay data to the gateway efficiently and help balance energy usage across the network. Therefore, direct transmission is energy-intensive and impractical for distant nodes, routing protocols establish multi-hop paths, where intermediate nodes or cluster heads relay data towards the gateway in an energy-efficient manner [43]. From there, it enters the Transport Layer, where wireless communication infrastructure (like base stations or IoT management platforms) securely transmits the data to cloud or centralized servers. Once received, the data reaches the Application Layer, where it is processed, analyzed, and visualized by specific applications—such as fire detection systems, smart transportation, or industrial automation—enabling real-time monitoring, alerts, and decision-making by end users. Thus, the routing efficiency from sensor nodes to the gateway is critical, as it directly influences network lifetime, scalability, latency, and the overall quality of service delivered to IoT applications [44,45,46].

Figure 3 depicts a layered IoT-based wireless sensor network in which sensed data is progressively transferred from field devices to cloud services. At the lowest level, the sensing layer consists of sensor nodes that monitor the environment; the source node forwards its readings over multiple hops through intermediate relays, with nodes A and B illustrating alternative forwarding points that may be chosen according to current link quality and residual energy. The dashed curve represents the active routing path from the source towards the sink node. Above this, the transport layer is composed of wireless gateways and base stations, shown as antenna symbols, which collect traffic from the WSN and relay it upward. At the top, an IoT management platform supervises connectivity, aggregates and processes the incoming data, and exposes it to application-layer services such as fire detection, smart transportation, smart grids, industrial automation, and cloud-hosted monitoring.

Table 2 shows the communication layers within IoT-enabled Wireless Sensor Networks, along with their respective roles, technologies, and routing involvement. Each layer contributes uniquely to the end-to-end data flow, beginning with the Perception Layer, responsible for sensing and digitizing environmental data through devices such as RFID tags, ZigBee modules, or LoRa nodes [42,48]. Although routing is not handled here, this layer initiates the data lifecycle. The Middleware Layer plays a bridging role by aggregating and semantically processing data, supporting interoperability through cloud-facing protocols like MQTT and CoAP [49]. Still, it has limited direct involvement in routing tasks. In contrast, the Network Layer serves as the core of routing activity. It manages multi-hop communication between nodes using energy-aware routing protocols such as LEACH, AODV, and RPL [50]. This layer is instrumental in determining optimal data paths, balancing load, and ensuring quality of service (QoS)—making it the primary focus for energy-efficient routing strategies in WSNs. Recent studies have emphasized optimization-based and AI-driven routing at this layer to prolong network lifetime and mitigate hotspot problems [51,52]. Finally, the Application Layer delivers insights and controls to end users via dashboards, APIs, and mobile interfaces, completing the data journey [53]. While routing responsibilities are centralized within the network layer, the coordination and efficiency of all layers are essential to sustaining robust and intelligent IoT-WSN operations.

### 3.2. Applications and Challenges in IoT-Based WSN

**Figure 4:**
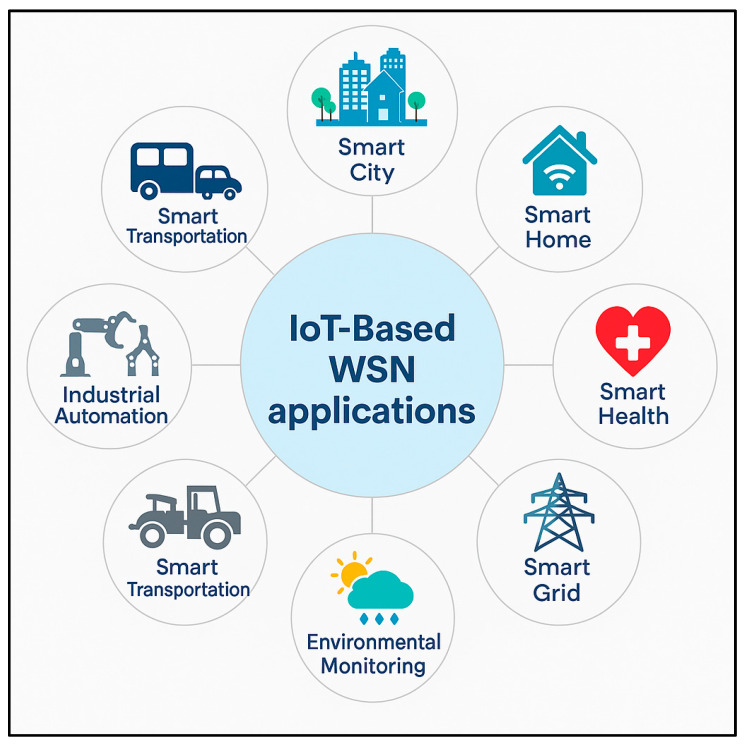
IoT-based WSN applications.

Wireless Sensor Networks (WSNs) have become indispensable enablers of the Internet of Things (IoT) landscape, supporting a wide range of real-world applications spanning critical domains such as urban infrastructure, healthcare delivery, energy management, and industrial processes. As shown in Figure 4, these diverse applications demonstrate the versatility and importance of WSN-IoT integration across smart cities, transportation systems, residential environments, medical facilities, electrical grids, environmental systems, and manufacturing facilities. In smart agriculture, for instance, platforms like SmartFarmNet deploy dense networks of soil sensors to monitor moisture, temperature, and nutrient levels, allowing farmers to optimize irrigation and crop management [54,55]. Similarly, in urban environments such as Barcelona’s Smart City project, WSNs are used extensively for traffic control, pollution monitoring, and energy optimization [40,56].

Healthcare is another domain where WSNs demonstrate immense potential; wearable devices, such as Vital Patch, continuously gather patient health metrics, enabling doctors to monitor critical conditions and intervene remotely proactively [57,58]. In the industrial sector, WSNs form the backbone of smart factories, facilitating predictive maintenance and equipment monitoring to enhance operational efficiency [59,60].

Furthermore, environmental protection initiatives, such as Japan’s *Earthquake Early Warning System*, leverage WSNs to detect seismic activities and issue alerts that can save lives [61]. These real-world deployments demonstrate the versatile role of WSNs in enabling intelligent systems that can respond autonomously to dynamic environmental and operational conditions.

**Figure 5:**
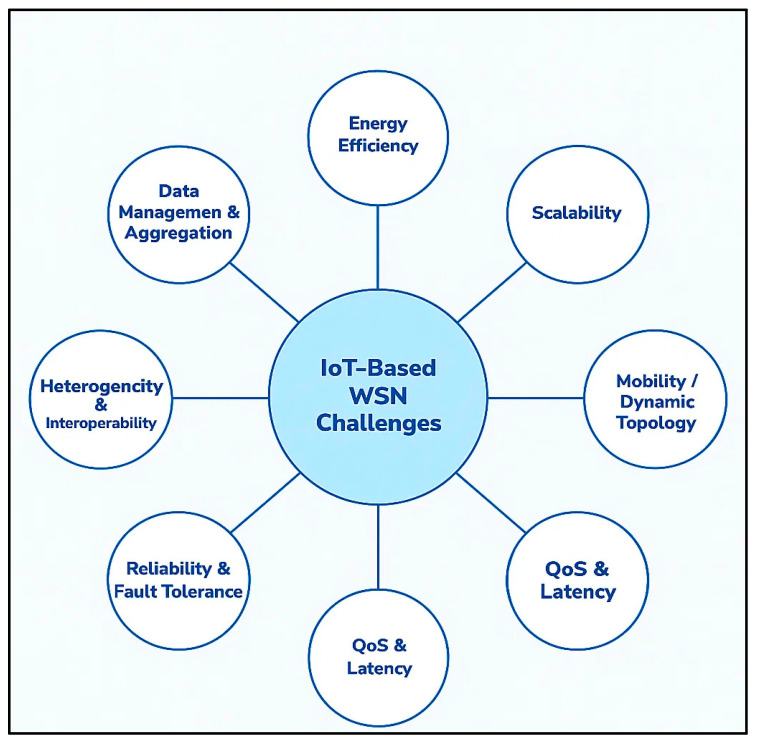
IoT-based WSN challenges.

However, despite their expanding footprint, WSNs in IoT environments continue to grapple with several persistent challenges that threaten their reliability and scalability. As shown in Figure 5, the most critical concern is energy efficiency, as many WSN nodes operate in inaccessible locations where frequent maintenance is impractical, making energy-aware design an urgent priority [50,62,63]. In large-scale smart city deployments, like those in Singapore, network scalability and congestion are emerging as significant hurdles as thousands of sensors compete for limited bandwidth [48,64]. Mobile WSN applications, including those utilising drone-assisted disaster monitoring, often encounter dynamic topology changes that complicate consistent data transmission. Healthcare applications relying on wearable sensors also highlight the challenge of ensuring high-quality, uninterrupted data streams in the presence of potential interference or device failure [58,65].

Security vulnerabilities remain a significant threat, particularly in industrial settings, where attacks on WSN infrastructures can lead to operational disruptions or data breaches [66,67]. Additionally, as future IoT-WSN applications move towards supporting autonomous vehicles, smart grids, and AI-driven environmental prediction, there will be an even greater demand for WSNs that are not only energy-efficient and resilient but also capable of real-time self-adaptation and intelligent threat detection. Addressing these challenges will be critical to unlocking the full transformative potential of WSNs in the evolving IoT landscape.

## 4. Routing Optimization for IoT-Based WSN: A Classification of Literature Review

**Figure 6:**
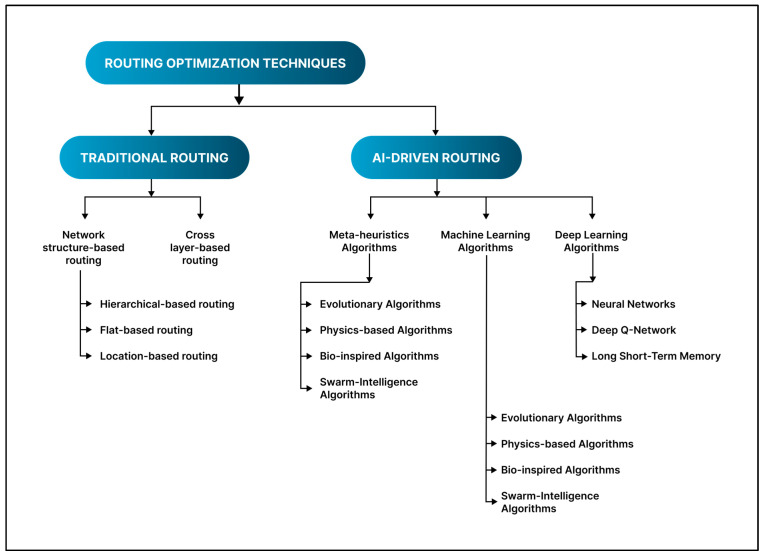
Taxonomy of routing optimization techniques.

WSN-IoT routing is a complex process that generates massive amounts of data. It determines the most efficient paths for data transmission while addressing challenges such as energy constraints, scalability, and dynamic network conditions [48,62,63]. In this routing, the network layer manages routing for incoming queries and monitoring messages [49]. In a multi-hop network, where source nodes cannot directly reach the sink node, intermediate nodes relay messages, and routing tables are employed to address this challenge effectively [51]. Numerous routing techniques have been formulated in WSN specifically for IoT contexts to enhance energy efficiency. We classify these strategies into two primary categories, as shown in Figure 6: the first being traditional ways and the second being AI-driven approaches. Conventional routing protocols, including flooding, shortest-path, and cluster-based techniques, depend on predetermined rules and heuristic algorithms to create communication pathways [44]. These methods frequently evaluate parameters such as hop count, energy levels, and established metrics to ascertain the optimal route. Nonetheless, they may encounter difficulties with fluctuating network circumstances, resulting in inefficient energy utilization, network longevity, and adaptability.

### 4.1. Traditional-Based Routing Techniques

In the context of networking and WSN, conventional routing techniques often employ predetermined, stationary, or semi-dynamic approaches that lack advanced learning-based or artificial intelligence-driven decision-making. Rather than using adaptive or intelligent optimization, these traditional methods depend on fundamental strategies such as shortest path selection, hierarchical routing, or flooding mechanisms.

#### 4.1.1. Network Structure-Based Routing Techniques

Among these, hierarchical routing protocols organize nodes into clusters, choosing cluster heads through various election algorithms to manage communication efficiently. Cluster heads then gather and transmit data from regular nodes to the base station, mitigating traffic congestion at higher communication layers [68,69]. By implementing clustering across multiple communication levels, these protocols significantly enhance network scalability by reducing the size of routing tables, thereby simplifying network management. Notable examples of hierarchical routing include the Cluster-Based Routing Protocol (CBRP) and the Low-Energy Adaptive Clustering Hierarchy (LEACH). Flat-based and location-based routing represent two distinct strategies in network routing, each characterised by unique decision-making mechanisms. Flat-based routing treats all nodes equally without hierarchical structuring, with every node independently maintaining a routing table and autonomously making decisions based solely on destination addresses and internal routing information. Examples include Distance Vector Routing Protocol (DVRP) and Link State Routing Protocol (LSP) [70]. In contrast, location-based routing decisions primarily depend on the geographical positions of nodes, rather than on traditional network topology or address-based methods. Here, nodes utilize location-aware devices, such as GPS receivers, to obtain geographic coordinates, enabling routing algorithms to select optimal paths based on physical location. The Geographical and Energy Aware Routing (GEAR) protocol exemplifies this approach [71].

To transmit data efficiently in WSNs, Chongtham et al. proposed an integrated version of LEACH that incorporates the A* search algorithm and introduces the energy threshold equation, which factors in the residual energy of nodes, prioritizing nodes with higher energy levels for selection as cluster heads [72]. This helps efficiently select the cluster head and optimizes the distance data packets travel from sensor nodes to their cluster heads, thereby reducing energy consumption in data transmission. However, no specific algorithm optimizes energy-efficient data transmission from the cluster head to the base station, which typically consumes significant energy. The disadvantage of cluster heads in WSNs is that a node far from the sink consumes more energy than a node closer to the sink.

To eliminate the long link between CH and sink, other authors proposed modifying the LEACH and PEGASIS approaches to enhance LEACH by incorporating PEGASIS’s chain formation strategy for data transmission; CHS forms a chain to relay data to a Leader Node (LN), which is the CH closest to the sink node [73]. The primary concept of the leader node (LN) involves collecting data packets from sensor nodes and then forwarding them to the sink node, thereby reducing energy consumption and extending the lifespan of the IoT Network. The problem with this approach is its limited parameters; however, it does not consider other important parameters, such as delay, throughput, and packet loss, which are also crucial for reliable transmission. The cost of CH is higher than that of ordinary nodes, so fairer CH selection becomes an integral part of the routing algorithm; otherwise, the nodes will die soon, resulting in a reduced lifespan of the WSN-IoT.

Another important issue is data transmission during poor link quality. Chen [74] integrated link quality into LEACH protocol for enhancements by evaluating the reliability and strength of connections during cluster head selection and data transmission. By choosing routes and cluster heads with higher link quality, the network reduces the likelihood of data retransmissions and minimizes energy expenditure on unstable paths. This adaptive selection mechanism ensures that nodes with robust communication links handle greater data forwarding responsibilities, thereby extending network lifetime and distributing energy consumption more evenly among nodes. The overall strategy results in improved energy efficiency for wireless sensor networks, with fewer wasted transmissions and more stable, optimized routing. However, this method may overlook nodes with better link quality, as it selects CHs solely based on energy levels, potentially leading to suboptimal network performance and increased transmission errors. Srinivas et al. asserted that selecting the shortest path can conserve energy in sensor nodes during WSN routing. CbCFRP utilized the Chimp optimization algorithm to determine the optimal routes for data transmission, thereby minimizing energy consumption and delay [75]. The author suggested that the Chimp fitness function is designed to track the shortest routes between the source and the destination. This protocol was evaluated and compared with existing methodologies using metrics such as throughput, delay, packet drop rate, and delivery rate to highlight its improved performance. In the quest for energy-efficient routing paths, certain sensor nodes may be left unattended where battery replacement is not feasible. To solve this problem, in 2023, Dogra et al. proposed an innovative region-based routing protocol that optimizes cluster head selection based on residual energy and utilizes multi-hop communication to reduce energy consumption and extend the network’s lifetime [76]. The network is divided into regions, and nodes are grouped into clusters within these regions to minimize energy consumption. The protocol selects new cluster heads based on their residual energy, ensuring that nodes with higher energy levels are more likely to become cluster heads. This approach is effective only in static networks where node positions remain fixed throughout the sensor nodes’ lifetimes. However, if nodes become mobile, it can negatively impact the performance of the WSN, particularly in terms of energy consumption and network stability.

#### 4.1.2. Cross-Layer Design Approach

The other classic routing protocol is a cross-layer design approach that enhances overall performance and efficiency by enabling interaction and optimization across different network protocol stack layers [77]. While traditional network architectures isolate communication tasks into distinct, independently operating layers, cross-layer design addresses the unique challenges of IoT environments—such as limited energy resources, varying network conditions, and real-time data processing—by allowing coordinated and adaptive optimizations across these layers [78].

To address routing challenges in WSN-IoT networks, particularly those related to dynamic network conditions, device mobility, and efficient resource utilization. To solve this issue, Jin-Woo Kim et al. proposed a Cross-Layer MAC/Routing Protocol for IoT networks, which improves path discovery and selection by using dynamic addresses assigned during network formation to identify optimal routes based on minimizing hop count [79]. Additionally, the protocol uses beacon frames at the MAC layer for time synchronization, which enhances communication efficiency and conserves energy. TSMR also includes association and reassociation procedures to support device mobility and network robustness. By integrating MAC layer information into routing decisions, the protocol considers channel quality and device proximity factors, resulting in improved path selection and reduced transmission errors and delays, thereby significantly enhancing network performance and reliability.

Another way to ensure reliability and deadline-sensitive data communication while maintaining QoS for both real-time (RTD) and non-real-time (NRT) traffics in WSN using CWSN-eSCPM routing protocol. This routing method integrates innovative strategies such as Proactive Node Management, Data-Centric Service Differentiation and Fair Resource Scheduling (DCSDFRS), Packet Velocity Estimation, Cumulative Congestion Estimation, and Dynamic Link Assessment to achieve reliability [80]. The protocol prioritizes nodes capable of meeting deadline-sensitive transmissions by estimating packet velocity. Dynamic link assessment and congestion estimation enable the selection of forwarding nodes that minimize delays and packet losses. Together, these mechanisms ensure the optimal selection of the Best Forwarding Node (BFN), addressing RTD and NRT data requirements and ensuring QoS through optimal resource allocation for all data types.

In long-distance transmission, a trade-off problem arises between energy efficiency and optimal data transmission in WSN-IoT networks, which Mahajan solves using a Nature-Inspired, algorithm-based Cross-layer Clustering (NICC) protocol [81]. This protocol uses the Bacterial Foraging Optimization (BFO) algorithm to select optimal sensor nodes for clustering and routing. The NICC protocol’s innovation lies in its cross-layer probability computation, where the fitness value of each sensor node is determined based on parameters from the network, physical, and MAC layers. This cross-layer approach calculates a fitness function to ensure a trade-off between energy efficiency and optimal data transmission, thereby enhancing both energy and QoS efficiency, which are critical for deploying smart farming solutions.

To maintain efficient routing, several factors must be considered, including efficient route discovery and path selection, estimating the required transmission power based on gathered data, and minimizing bit rate errors, all of which are crucial for enhancing the reliability of the WSN-IoT network. All these issues are discussed and solved by the CLEERDTS approach, which integrates cross-layer information from the network, MAC, and physical layers [82]. The network layer uses node location and SNR values for optimal route selection, while the MAC Layer estimates transmission power based on topology. The physical layer adjusts the transmission mode for reliable communication. If an optimal route is unavailable, the model prioritizes relay nodes with high residual energy and adjusts power to reduce Bit Error Rates (BER). Simulation results demonstrate that CLEERDTS enhances energy efficiency, packet delivery, error rates, and throughput, making it an ideal solution for applications that demand high data accuracy and minimal energy consumption.

To address the computational overhead issues in WSN-IoT routing, the extra processing, memory, and energy burden placed on sensor nodes for executing complex tasks such as data aggregation, routing decisions, security operations, or optimization algorithms like Particle Swarm Optimization (PSO). Due to the limited CPU power, memory, and battery life of sensor nodes, the heavy computational demands can quickly drain resources, shorten network lifetime, and impact real-time responsiveness. Basically, this paper contributes to the ECCM approach by integrating fog computing with a cross-layer approach and utilizing PSO for intelligent cluster head selection. The ECCM algorithm applies a sensing event-driven mechanism to project fog nodes onto the sensing layer [83]. This forms a potent virtual control node that fundamentally changes how clusters are managed, and data is routed within the network. ECCM facilitates the distributed clustering of event-field nodes by elevating the control procedure of the cluster-based routing protocol to the fog layer. This approach used fog computing to perform computations and manage the clustering process, thus offloading significant computational tasks from the sensor nodes to the fog nodes. Using the ECCM approach, the author introduced the particle swarm optimization (PSO) algorithm to elect a group of optimal nodes as cluster heads. This selection is made without competition overhead, significantly reducing and balancing the network’s energy overhead. This strategic move aims to prevent the rapid exhaustion of node energy, thus extending the network’s lifetime.

A variety of methods have been advanced for routing and clustering in wireless sensor networks, each authored by different researchers and bearing distinct strengths and weaknesses. Arunkumar’s hierarchical protocol enhances security and prolongs node lifespan through cluster structuring, though the elevated security levels may introduce increased delays and expose the system to power depletion in cluster leaders, potentially hampering network throughput and overall reliability [84]. Prince, Kumar, and Singh’s multi-level clustering and predictive routing mitigate energy inefficiencies tied to hotspot formation, leveraging layered predictions for better distribution, but these hierarchical arrangements contribute to organizational overhead and latency, with network robustness still sensitive to cluster head failures [85]. Moussa, Khemiri-Kallel, and El Belrhiti El Alaoui employ a fog-assisted hierarchical approach for IoT-enabled sensor applications, which reduces both energy usage and transmission distance via intermediary fog devices, but this strategy’s effectiveness is dependent on the reliability and computational load of fog nodes; any disruptions at this level can amplify latency and degrade fault resilience, particularly under heavy traffic conditions [86]. These works collectively showcase how improved energy utilization, enhanced security, and adaptive routing are achieved with advanced clustering and hybrid approaches, yet the protocols still face critical limitations relating to latency overhead, vulnerability to key node failures, and throughput challenges in real-world scenarios.

For mobile IoT application, Al-Sadoon, Jedidi, and Al-Raweshidy formulated a dual-tier cluster-based routing strategy, aiming to improve adaptability and conserve battery life during node mobility; however, this method encounters increased latency during cluster handovers and remains vulnerable to throughput drops in highly mobile conditions [87]. Cherappa and colleagues advanced energy-efficient routing using Adaptive Swarm Firefly Optimization (ASFO) combined with a cross-layer design, which efficiently organizes clusters and adapts transmission routes, but the underlying optimization algorithms can add computation delays and have limited resilience in dynamic environments [88]. Sarwesh and Mathew’s cross-layer protocol with a weighted sum approach seeks to extend device longevity in smart city scenarios by balancing multiple network factors, though as density increases, delays mount and large-scale failures pose a challenge for fault management [89]. Renaldo Maximus and Balaji pioneered fuzzy logic integrated with Barnacle Mating Optimization, enabling hybrid clustering and cross-layer routing; their solution offers strong energy savings and minimized delays in stable networks, but the system’s adaptation time can grow under rapid changes, making it moderately sensitive to cluster leader disruptions [90]. Mahajan, Badarla, and Junnarkar’s CL-IoT protocol exploits cross-layer techniques for intelligent manufacturing and smart farming, promoting reliable throughput and low latency in industrial settings; yet its scalability is limited outside controlled deployments and its fault recovery strategies falter under severe environmental stress [91]. Lastly, Tandon and Srivastava [92] contributed a location-aware, secure cross-layer protocol for IoT, where energy efficiency is achieved by using node positions and security management, but network delays increase with dense topologies and fault tolerance stays constrained during security attacks or hardware failures.

In Table 3, we observe that the traditional routing algorithms such as hierarchical protocols including LEACH, PEGASIS variants, and clustering approaches continue to face significant limitations in terms of energy efficiency, scalability, and reliability for IoT networks. These solutions often rely on fixed or static configurations for cluster formation and address allocation, resulting in uneven energy depletion, increased control overhead, and poor adaptability when the network topology or traffic patterns change. Their cross-layer enhancements may improve certain performance metrics. Still, they do not fully address the challenges posed by dynamic node mobility, highly dense deployments, or application-specific QoS requirements typical in real IoT scenarios. Limitations also persist in ensuring robust connectivity during frequent node association/reassociation, managing control overhead efficiently, and supporting seamless integration across heterogeneous network layers. As a result, these protocols can suffer from increased packet loss, excessive delays, and network partitioning, especially when the network scales or faces unpredictable conditions, making them insufficiently energy-efficient, scalable, and reliable for the evolving demands of IoT environments.

### 4.2. AI-Driven Routing Algorithms

Traditional routing methods typically find it difficult to accommodate dynamic network conditions, energy limits, and scalability issues, such as those found in Wireless Sensor Networks (WSNs) and IoT systems, which get more complicated. By applying meta-heuristics routing, machine learning (ML), deep learning (DL), and reinforcement learning (RL), AI-driven routing algorithms produce intelligent, predictive suggestions for optimizing routing paths. These systems constantly modify routing techniques to improve energy efficiency, fault tolerance, load balancing, and Quality of Service (QoS), analyzing real-time network characteristics and projecting link quality. Through ongoing learning and adaptation, AI-based routing solutions offer greater flexibility, improved network longevity, and enhanced performance compared to traditional rule-based methods.

#### 4.2.1. Meta-Heuristics Routing Algorithms

To address limitations of static protocols, metaheuristic algorithms have been increasingly applied to optimize routing decisions. Genetic Algorithms (GA), Particle Swarm Optimization (PSO), Ant Colony Optimization (ACO), and Artificial Bee Colony (ABC) have been widely explored for cluster head selection, relay node identification, and multi-hop path optimization [93]. These methods improved energy balancing and network lifetime compared to classical techniques; however, their convergence speed and parameter sensitivity often restricted their real-time applicability. Hybrid variants, such as PSO-LEACH or Firefly-assisted clustering, demonstrated better adaptability but introduced additional computational overhead at resource-constrained nodes. Metaheuristic algorithms have been widely adopted to optimize cluster-head (CH) selection, relay node identification, and path formation in IoT-WSNs. These techniques are attractive because they can efficiently search large spaces while considering multiple objectives such as residual energy, distance, and link quality.

One Key reason for NIAs’ popularity is their ability to tackle various challenging engineering problems effectively. These problems may include optimization tasks in various domains such as logistics, manufacturing, telecommunications, finance, and healthcare. By mimicking the natural behaviors of organisms or natural phenomena, NIAs offer innovative and efficient approaches to problem-solving for network optimization. Leach is the basic routing protocol used for data transmission in WSNs [94]. However, cluster head selection becomes a major challenge when transmitting data from source to destination, because LEACH selects cluster heads randomly, which can lead to uneven energy consumption. Nodes with low remaining energy might be chosen as cluster heads, leading to their early depletion and reducing the overall network lifetime.

To minimize energy consumption during data transmission and to enhance network longevity, the Orphan-LEACH (O-LEACH) protocol has proposed and developed a hybrid optimization approach using Simulated Annealing with Lightning Search Algorithm (SA-LSA) and Particle Swarm Optimization with LSA (PSO-LSA) [95]. The SA-LSA algorithm uses the Local Search Algorithm (LSA) as its core, enhanced by Simulated Annealing (SA), to optimize a population more effectively through a novel two-point crossover method. Although LSA converges quickly, it struggles with multimodal optimization, so the authors also use PSO. PSO finds solutions rapidly but may not guarantee the global optimum in complex spaces. By integrating PSO with LSA, the hybrid approach efficiently improves cluster head selection and path routing, reducing energy consumption and extending network lifetime. Tuning the balance between exploration and exploitation in PSO is key to maximizing these gains.

Ant colony optimization works well in dynamic networks to find the optimal path. However, optimizing factors such as transfer probability and pheromone concentration in IoT applications can cause difficulty in finding reliable paths in real-time applications. To solve these issues, Han proposed an approach to improve the ant colony algorithm. They make ants smarter by adjusting and updating pheromone levels on different paths, emphasizing the importance of these paths during the search process [96]. It addresses challenges like energy constraints and dynamic network topologies and enhances data transfer quality, supporting real-time applications. However, the approach has limitations, such as potential inefficiency with increasing problem complexity and sensitivity to parameters like pheromone evaporation rate and ant numbers, making parameter optimization challenging and time-consuming.

Efficient clustering is the key challenge in the LEACH protocol for the WSN-IoT network, which is solved by combining Aquila Optimizer (AO) and Firefly Algorithm (FA) to optimize clustered routing and energy consumption [97]. In the paper, the author evaluates the effectiveness of this hybrid method concerning the usage of energy, network throughput, packet delivery ratio, and other crucial metrics. This approach is compared with other existing methods, demonstrating its effectiveness in addressing the challenges of energy-efficient routing in IoT networks. However, other factors like sink distance, intra-cluster distance, node degree, energy level, and priority factors can also greatly impact the enhancement of sensor node energy for reliable routing.

Existing approaches, such as the Grey Wolf Optimization (GWO) algorithm [98], have been developed to tackle this challenge by considering factors like sink distance, intra-cluster distance, node degree, energy level, and priority factors. While GWO enhances the Quality of Service (QoS) by employing a novel fitness function for CH selection and a cost function for QoS-aware relay node selection, it faces challenges such as increased communication overhead and delays in dynamic environments with mobile nodes. To address these limitations, the EOR-iABC strategy offers an improved solution for optimal path selection, ensuring reliable and scalable routing [99]. This method periodically selects energy-efficient CHs using an advanced artificial bee colony algorithm incorporating crossover and mutation processes. By combining the roles of employee and onlooker bees, the algorithm enhances local search strategies, reduces delay convergence, and increases the likelihood of selecting high-fitness nodes as CHs. The Grenade Explosion Method (GEM) and the Cauchy operator also expand the search beyond local areas, improving global optimization. However, this approach introduces computational complexity and overhead challenges in large-scale networks and diverse IoT environments. Despite these trade-offs, the protocol improves network performance by optimizing CH selection and data collection efficiency.

One of the persistent challenges in these networks is the high cost associated with establishing optimal paths from the source to the sink, particularly in dynamic and resource-constrained environments. Traditional routing techniques often struggle to strike a balance between energy efficiency and computational overhead, leading to increased execution times and inefficient data transmission. The author developed a CUCKOO-ANN-based optimization technique that combines the CUCKOO algorithm with Artificial Neural Networks (ANN) to create a more effective, reliable, and energy-efficient solution [100]. The CUCKOO search algorithm balances global and local optimization through random egg placement, selection of best nests, and a discovery phase. These best nests are then processed by an ANN to select energy-efficient routes from source to sink. This hybrid method combines effective nonlinear problem-solving and fast parallel processing, optimizing data transmission and reducing execution time in energy-constrained networks.

By observing Table 4 one can find that transitioning from single-hop to multi-hop communication between CHs and BS is considered an energy-saving approach. Multi-hop communication uses intermediate nodes such as relay nodes to create an efficient path to the BS. However, hybrid optimization techniques, especially those using swarm intelligence, introduce additional complexity in CH selection, particularly with the exploration and exploitation trade-off. Several cluster-based models depend on multi-hop and multipath routing, but the need for frequent coordination and synchronization, particularly in mobile sink environments—leads to increased latency and routing overhead. Moreover, fault tolerance and reliability-enhanced schemes, although robust in managing errors and failures, often present energy inefficiencies when managing high-energy consumption routines or resource-intensive fault detection mechanisms. Load-balancing and multi-sink optimizations solve specific issues such as hotspot formation and event detection yet inherently depend on precise location data and clock management, making them susceptible to synchronization delays. Overall, while each method attempts to reasonably optimize energy use and resilience, the operational burden associated with complex selection rules, agent bloat, or real-time processing requirements remains a fundamental challenge that consistently impacts delay, scalability, and sustained network performance.

#### 4.2.2. Machine Learning-Based Routing Algorithms

Machine Learning-based routing uses historical data and real-time network dynamics to make informed routing decisions that dynamically adapt to changing network conditions. One key advantage of using ML in IoT routing is its predictive capability. By analyzing historical data, ML models can forecast future network conditions, such as congestion points, potential link failures, or node energy depletion. This foresight enables proactive routing adjustments to prevent potential issues from impacting network performance. Machine learning is now widely utilized to design adaptive and efficient routing protocols for IoT networks, helping to address challenges such as energy efficiency, scalability, and dynamic topology changes [115].

Basically, ML approaches come under three learning: Supervised, unsupervised and reinforcement learning. Supervised Learning is used for predictive routing based on labelled historical data [116]. Models such as Support Vector Machines (SVM) and Neural Networks are trained on features like traffic volume and link status to predict the most optimal route paths. Reinforcement Learning (RL) routing decisions are made based on the reward received from the network environment [117]. Algorithms like Q-learning adaptively learn the most efficient routing strategy by maximizing the cumulative reward, which could be based on metrics like reduced energy consumption or improved latency. Unsupervised Learning Techniques [118], such as clustering, are used to group nodes or data flows with similar characteristics. This can be useful for identifying natural hierarchies in network structures or for traffic differentiation in routing.

A significant obstacle in IoT-oriented networks is coping with constant changes in network structure and energy resources, leading to less efficient routing and increased power drain. Traditional routing protocols are often unable to adapt to these shifting dynamics, which results in limited scalability and faster depletion of energy. To address these issues, protocols like EER-RL [119] have adopted adaptive reinforcement learning strategies. Here, each node independently learns routing choices that conserve energy and ensure data delivery by processing local and global network factors. The protocol updates node routing behavior dynamically based on feedback, streamlining communication paths and reducing overall energy use. This leads to longer network operation, improved alignment to real-time conditions, and better scalability than conventional methods.

A significant challenge is the unpredictability of node failures, which can lead to data loss, decreased network reliability, and QoS deterioration. Existing methods often react to failures rather than prevent them, resulting in inefficient data transmission and higher energy consumption. In response to this challenge, Sharma et al. introduced a novel unsupervised machine learning model based on the Local Outlier Factor (LOF) algorithm [120] to predict imminent node failures, allowing proactive rerouting for improved reliability. This is combined with a Q-learning-based reinforcement strategy that adaptively optimizes paths, achieving energy efficiency and sustained network performance in IoT deployments.

Majumdar et al. [121] address traffic congestion prediction by integrating IoT devices with machine learning techniques to forecast congestion propagation. Specifically, using Long-Short-Term Memory (LSTM) networks allows for identifying temporal patterns in traffic data, enabling accurate anticipation of congestion. This predictive capability supports the development of smart traffic management strategies, guiding road users to avoid congested areas. Reducing traffic density and associated air pollution promotes sustainable urban mobility and efficiently mitigates congestion in urban environments.

Data collection is a very tedious and energy-draining task for sensor nodes in dynamic environments. So, Krishnan et al. [122] proposed a reinforcement learning model using a mobile sink for dynamic data routing in WSNs. The mobile sink adjusts cluster heads based on energy levels, balancing energy consumption across the network. This approach overcomes the energy hole problem associated with static sinks, extending network lifetime and improving data collection reliability and efficiency by adapting to the dynamic network environment. Several authors now use decentralized approaches in machine learning. As we can see in this paper, Soltani et al. [123] proposed a multi-agent reinforcement learning framework where distributed agents cooperatively refine their routing policies based on network conditions, resulting in adaptive energy consumption and longer network lifespans. Godfrey and colleagues [124] take a similar approach by embedding reinforcement learning into software-defined wireless sensor networks, enabling centralized controllers and edge nodes to coordinate route selection dynamically to enhance energy balance and system resilience. Li and Ai [125] introduce a hybrid clustering protocol using Tabu Search and Ant Colony Optimization, which enables efficient cluster head selection and path optimization, ultimately distributing communication load more evenly and reducing the risk of premature node failure. Shin and Lee [126], as well as Razooqi and Al-Asfoor [127], demonstrate the efficacy of swarm intelligence and bio-inspired optimization for decentralized path discovery and load distribution, allowing the network to rapidly adapt to changing topologies and node failures.

Al-agar et al. [128] and Norouzi and Zaim [129], highlight the use of genetic algorithms for optimizing clustering and routing, minimizing redundant communication, and maximizing coverage with minimal energy draw. These solutions excel at managing fault tolerance and maintaining throughput in complex, multi-hop environments by continually refining routing tables with evolutionary search strategies. Kamel, Yang [130], and Hu [131] each explore Particle Swarm Optimization (PSO) combined with fuzzy logic enhances energy-efficient clustering and routing in WSNs, extending network lifespan and reliability. These hybrid approaches use fuzzy rules based on factors like residual energy and node proximity to select optimal cluster heads and routing paths. While effective, challenges remain in managing computational overhead, complex parameter tuning, and maintaining performance in dynamic networks, necessitating ongoing research for practical large-scale deployments.

According to Bzai et al. [115], ML in IoT environments is commonly examined through three main lenses: data, applications, and industry. The data perspective emphasizes managing the diverse and often complex types of IoT data, including structured, semi-structured, and unstructured formats from various sensors, using processes such as preprocessing, feature extraction, and noise filtering to build reliable predictive models. From the applications viewpoint, ML is adapted to meet the needs of different IoT domains such as smart cities, healthcare, agriculture, and autonomous vehicles, where it addresses challenges like latency, energy efficiency, and network dynamics to enhance decision-making and automate operations. The industry perspective concentrates on the real-world deployment of ML-powered IoT solutions, focusing on issues such as scalability, security, interoperability, and compliance, while leveraging emerging architectures like edge and fog computing to optimize performance and resource utilization. This comprehensive classification helps understand how ML facilitates intelligent, efficient, and secure IoT systems across a variety of settings.

In Summary, ML has played a transformative role in shaping wireless sensor and IoT network routing, introducing adaptive decision-making and improving network responsiveness to changing conditions. Despite these benefits, its real-world deployment often encounters important technical hurdles. Processing complex models or updating learned parameters can demand significant computational power and memory, particularly challenging compact sensor devices with limited resources. As a network expands or faces faster traffic shifts, maintaining synchronization and managing frequent data exchanges can lead to increased delays and potentially disrupt swift routing updates. Additionally, machine learning methods typically need substantial amounts of relevant data for effective training, and repeated fine-tuning as network dynamics evolve—tasks that add to operational complexity. As a result, while such algorithms boost overall system flexibility, energy savings, and resilience, their effectiveness may be constrained in high-density, rapidly changing, or weakly resourced settings. Designing practical, scalable, and lightweight ML models remains a crucial direction for further research and engineering in WSN-IoT deployments.

#### 4.2.3. Deep Learning-Based Routing Algorithms

Deep learning plays a vital role in solving routing issues in WSN and IoT systems. Using multi-layer neural models, it automatically learns patterns from sensor data and makes smart routing decisions. Integrated with IoT networks, deep learning methods like DNNs and DRL optimize routes, predict link quality, manage energy, and prevent congestion—making networks more adaptive, efficient, and reliable in dynamic environments.

Recent research shows that hybrid models, such as those blending deep reinforcement learning (DRL) with graph neural networks (GNN), can optimize network coverage, extend network lifetime, and significantly improve both throughput and latency—outperforming legacy methods like particle swarm and classical machine learning algorithms. Paulraj and Deepa [132] propose a neuro-fuzzy routing mechanism that uses historical and real-time network parameters to form dynamic clusters and improve data routing, increasing energy retention and throughput while reducing delay and node mortality; the limitations lie in sustaining performance when confronted with variable network sizes and abrupt traffic bursts, requiring continuous parameter adjustment. Pushpa et al. [133] introduce deep reinforcement learning with graph neural networks to dynamically optimize sensor placement and maximize coverage, enhancing efficiency and scalability by leveraging the spatial dependencies in node interactions; the drawback is the significant training overhead and the complexity of integration when adapting to rapidly evolving network states. Priyadarshi et al. [134] present an AI-based modular routing framework that blends reinforcement, supervised, and swarm-based learning to improve packet delivery ratio, latency, and energy metrics, with demonstrated gains in adaptability and reliability; however, the constraints on resource-constrained sensor nodes and the challenges of real-world scalability persist, such as security vulnerabilities and system maintenance in dynamic deployments. Collectively, while these deep learning approaches offer superior performance in terms of optimizing key parameters, their limitations stem mainly from the demands of computational resources, training complexity, network heterogeneity, and the need for robust fault tolerance under highly variable operating conditions.

Convolutional Neural Networks (CNNs), a type of supervised deep learning model, have been applied in wireless sensor network routing to efficiently process real-time sensor node data, such as signal strength, packet loss, node energy, and congestion levels. By transforming this raw input into concise feature maps through layered convolutions and pooling, CNNs capture both spatial relationships and signal patterns across the network, enabling intelligent prediction of link quality, optimal paths, and energy consumption. A notable example is the protocol by Guru Moorthy et al. [135], where Deep CNNs are used for predicting energy levels, while the Bald Eagle Assisted Sparrow Search Algorithm (BEA-SSA) selects cluster heads based on multi-factor analysis. This approach also integrates security assessment by analyzing trustworthiness and signal strength (RSSI) to maintain data integrity and minimize vulnerabilities. Overall, the system is designed to reduce energy usage, transmission delay, and improve overall routing reliability by adaptively selecting robust routes and cluster heads in response to live network status—leading to greater sustainability and performance in energy-constrained sensor deployments.

To improve energy efficiency and data collection in wireless sensor networks, Saravanan K. et al. [136] introduced a Rank-Based Path Planning approach combined with Recurrent Neural Networks (RPP-RNN), where the mobile sink prioritizes visiting hotspot nodes with the highest energy consumption, packet volume, and data transfer rank. Instead of accessing all nodes, the sink collects data from those most impactful, reducing overall energy use. The integrated RNN model dynamically predicts the optimal travel path considering distance, pause time, and hotspot distribution, leveraging sequential learning from previous movements to optimize future sink trajectories and maximize network lifespan.

By integrating the Twin Delayed Deep Deterministic Policy Gradient (TD3) algorithm with a Graph Neural Network (GNN), Zhang et al. [137] address problems like fluctuating topology and limited energy reserves. Rather than relying on static rules, their system learns continuously from real-time network data and adapts routing policies by balancing energy consumption, throughput, and latency. The TD3 component makes nuanced routing decisions over continuous actions, while the GNN accelerates learning by modeling node relationships and traffic patterns. To assign link weights, the protocol leverages Dijkstra’s algorithm, ensuring optimal path choices. This integration provides dynamic, data-driven route optimization, but introduces complexity and computational cost, highlighting a trade-off between improved adaptability and increased resource requirements.

Table 5 shows that intelligent routing methods for WSN and IoT usually succeed in lowering energy use and often help reduce delay and support larger networks, but this comes with added algorithmic complexity and limited suitability for highly dynamic, low-power deployments. Reinforcement-learning and deep-learning based schemes, such as multi-agent Q-learning, DOS-RL, DRL combined with GNNs, GTD3-NET and other modular AI designs, are able to react to changing conditions and, in some cases, forecast link behavior; however, they demand considerable processing capability, memory, and training data, which makes implementation on simple sensor nodes difficult. Approaches inspired by biological processes or soft computing, including GA, PSO, quantum PSO, neuro-fuzzy routing, and CNN assisted by BEA-SSA, typically offer strong gains in energy conservation and reasonable delay in static or mildly dynamic scenarios, yet they still require careful parameter adjustment, react slowly when the topology changes quickly, and impose coordination overhead on cluster heads and relay nodes. 

In Summary, specifically, for RL-based routing, we now emphasize that while RL algorithms enable adaptive and robust decision-making in highly dynamic IoT networks, they are often hampered by high convergence times and significant memory requirements, especially during the training phase. This leads to increased energy consumption and may be impractical for resource-constrained sensor nodes, as supported by recent studies where substantial computational and storage costs have been observed during model learning and policy updates. Similarly, metaheuristic approaches, although powerful for cluster head selection and energy balancing, can experience long runtimes due to the need for population-based iterative searches. These algorithms may struggle to scale efficiently and, without careful parameter tuning, can converge prematurely or get trapped in local optima. Regarding cloud and edge-based architectures, we discuss the trade-off between enhanced computational capacity and increased latency or energy overhead due to frequent data transfers, which must be weighed against the benefits of centralized processing. Another insight for deploying sensors in real-world applications is to determine the network size and the number of sensor nodes, as these factors influence routing protocol selection for system implementation. In small-scale WSNs with relatively few sensor nodes (for example, under several hundred), simpler flat routing methods or even direct single-hop transmission may be sufficient, especially when all nodes are within communication range of the sink. As the network grows larger with thousands of nodes, the scalability becomes a major concern. In these scenarios, hierarchical or cluster-based routing protocols are generally preferred because they help manage energy consumption, organize data forwarding efficiently, and handle increased network overhead caused by many-to-one traffic. For very large networks, advanced methods such as ML/DL are used when your network is dynamic, complex, or you require intelligent, self-adaptive, and multi-objective routing that classical protocols cannot achieve efficiently.

#### 4.2.4. Quantitative Comparison of AI-Based and Conventional Routing Protocols

This subsection provides a quantitative comparison of ten representative routing protocols for IoT-enabled WSNs, encompassing both conventional and AI-based schemes. The analysis is designed to demonstrate how AI-driven routing protocols outperform non-AI approaches across the key performance metrics. The performance evaluation of routing protocols was conducted using ns-3 network simulator. The simulation deployment consisted of 100 sensor nodes randomly distributed across a 1000 × 1000 m^2^ geographical area, with the sink node positioned at the center coordinates (500, 500) m. Each sensor node was initialized with 1.0 Joule of energy, simulating battery-constrained devices typical of real-world IoT-WSN deployments. These simulation parameters enable realistic evaluation of routing protocol performance in terms of energy efficiency, network lifetime, packet delivery ratio, and latency across diverse network topologies and traffic patterns encountered in practical IoT-WSN applications.

Table 6 illustrates the quantitative performance benchmark of IoT based WSN energy-efficient routing protocols which highlight the superior capabilities of AI-driven techniques over traditional methods. The classical protocols like LEACH and PEGASIS exhibit high energy consumption (85 mJ and 65 mJ per round, respectively), longer latency (120–140 ms), and shorter network lifetimes (1200–1800 rounds), with no support for mobility. In contrast, metaheuristic approaches such as PSO-LEACH and AO-FA Hybrid significantly reduce energy usage (55 mJ and 48 mJ), improve PDR (94–97%), and extend network lifetime up to 3500 rounds. Regarding Machine learning-based EER-RL achieves further gains with only 42 mJ energy consumption, 85 ms latency, and 96% PDR. Deep learning protocols show even stronger performance, whereas CNN-BEA-SSA and RPP-RNN consistently deliver high throughput (50 kbps), low latency (88–95 ms), and PDRs around 97%, with network lifetimes exceeding 3300 rounds. Among all, DRL-GNN (TD3) stands out as the most efficient protocol, recording the lowest energy consumption (35 mJ), highest throughput (52 kbps), lowest latency (72 ms), highest PDR (98%), and the longest network lifetime (4100 rounds), while also supporting mobility.

**Figure 7:**
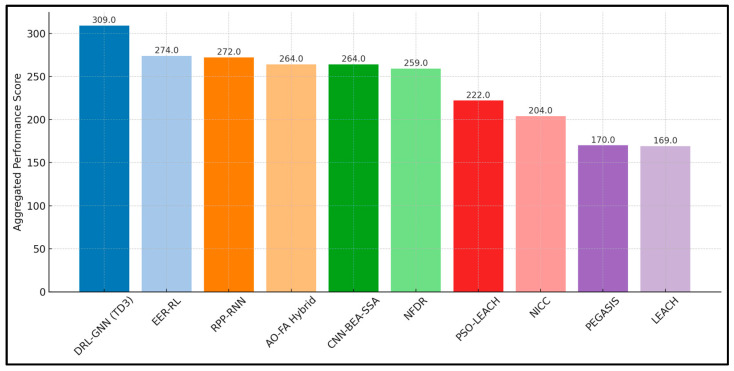
Overall performance comparison of routing protocols.

The bar chart in Figure 7 presents a comparative performance analysis of ten route-optimization techniques for IoT-based WSNs, where each bar represents an aggregated score derived from energy consumption, network lifetime, latency, throughput, and PDR. The highest score is achieved by DRL-GNN (TD3), because deep reinforcement learning combined with a graph representation of the network enables this protocol to continuously adapt routes to real-time topology, traffic load, and residual energy, which jointly minimizes energy use and delay while maximizing reliability and throughput. EER-RL, RPP-RNN, AO-FA Hybrid, and CNN-BEA-SSA follow closely; as AI-driven methods based on reinforcement learning, recurrent neural networks, and metaheuristic optimization, they learn or search for energy-balanced, high-quality paths and thus provide longer lifetimes and better QoS than fixed-rule designs.

In contrast, classical protocols such as LEACH, PEGASIS, and NICC obtain the lowest aggregated scores because they use static or probabilistic heuristics for cluster-head selection and routing, which leads to unbalanced energy depletion, higher average delay, and shorter network lifetime when the network becomes large or highly dynamic. Although these non-AI schemes are simple in operation and low-overhead, they lack the ability to react intelligently to changing traffic patterns, mobility, and heterogeneous node states, consequently their overall performance saturates well below than that of learning-based approaches. As shown in Figure 7, the bar chart highlights that AI-based and hybrid deep-learning routing protocols deliver substantial advantages in achieving energy-efficient, reliable, and scalable communication for modern IoT-WSN deployments.

## 5. Open Research Problems and Future Directions

The rapid evolution of IoT wireless sensor networks demands adaptive, intelligent, and holistic routing solutions that go beyond current methodologies. Building on the gaps identified in the literature, several promising future research directions and open opportunities are outlined below for advancing energy-efficient routing with AI and cross-layer innovation:

### 5.1. Open Research Problems

In this section, we list and describe five open research problems based on their importance, focusing on technological maturity, research challenges, and resource requirements.

(i) Limited Adaptivity and Scalability in Traditional and Hybrid Routing Protocols

Traditional and hybrid routing schemes in WSN and IoT networks often operate under fixed paradigms or pre-established conditions, making them ill-equipped for rapidly changing network landscapes. As systems scale in size or undergo frequent topology changes, these protocols struggle to reconfigure routes, balance energy loads, and manage increased communication overhead [141]. The result is often reduced network longevity and diminished throughput, as fixed rules cannot accommodate unforeseen events or large deployments. Greater adaptivity—in the form of self-organizing, real-time route selection—remains a crucial, yet unresolved, requirement for future protocols.

(ii) Inadequate Security, Robustness, and Fault Tolerance

Security and reliability are central to the effective operation of sensor networks. Most current protocols fail to address threats from within the network, such as malicious nodes or sudden device failures, relying instead on basic keys or simple trust metrics. These measures are usually insufficient against targeted attacks and can leave the network vulnerable to data interception or misinformation [142]. Moreover, recovery mechanisms for network faults and disruptions [143] are often limited, resulting in service interruptions and compromised data integrity when nodes are compromised or links go down.

(iii) High Overhead and Complexity in Bio-Inspired Cluster-Based Protocols

Bio-inspired routing techniques, including those that mimic swarm or evolutionary behaviors, hold promise for dynamic optimization, but their practical deployment can introduce substantial operational costs. These protocols typically rely on complex agent coordination, iterative optimization processes, and frequent updates, which demand more processing power and memory than many sensor nodes can provide [144]. This added complexity leads to higher energy drains, increased delays, and can slow down network adaptation, especially when operating on resource-constrained hardware.

(iv) Lack of Multi-Objective Optimization and Real-World Validation

Existing routing designs often focus on optimizing a single goal, such as energy usage or data delivery, without considering how multiple conflicting needs—like latency, load balancing, and security—interact in practical settings. Furthermore, many proposed protocols are only evaluated through simulation or limited lab tests, failing to account for unpredictable challenges in real-world deployments. Progress in this area requires integrating multi-objective optimization frameworks and conducting extensive field testing to verify that solutions hold under diverse, unstructured environments [145].

(v) Energy Hole and Network Fairness

A recurring challenge in cluster-based and hierarchical routing protocols is the uneven depletion of energy around certain nodes, particularly those near sinks or acting as cluster heads. This “energy hole” phenomenon causes premature failures, breaks connectivity, and reduces overall network lifespan. Moreover, protocols that do not fairly distribute communication and computation tasks among nodes can exacerbate this problem, leading to unreliable service and diminished performance in the network core [146].

### 5.2. Future Research Directions

In this section, we discuss five future research directions based on their importance, technological maturity, and research challenges.

(i) Advance Adaptive Routing with Context-Aware Deep Reinforcement Learning: Developing routing protocols that use deep reinforcement learning (DRL) integrated with context-awareness offers a pathway to networks that self-adjust to node mobility, fluctuating energy levels, and unpredictable traffic patterns. DRL agents can learn optimal routing actions through continuous interaction with the live network, adjusting strategies as environmental and operational variables change. Context-aware extensions use real-time sensor or environmental inputs—such as node location, link quality, and energy state—to further customize routing [147]. This approach can achieve a higher degree of adaptivity and scalability, allowing large and dynamic networks to maintain stability, efficiency, and prolonged operation, even as operating conditions shift rapidly.
(ii) Integrate Lightweight AI-Driven Security Features and Predictive Fault Detection: Enhancing security and resilience with lightweight, embedded AI modules presents a promising opportunity. By training miniaturized models at the edge or node level, networks can identify abnormal patterns, suspicious traffic, or potential failures in real time with minimal energy or computation costs. Such AI-driven systems can flag intrusions, trigger automatic rerouting, or isolate compromised nodes, thereby minimizing network disruption and maintaining data integrity. Predictive maintenance and anomaly detection backed by on-device intelligence can greatly improve fault tolerance and reduce repair or maintenance overhead in resource-constrained settings [148].
(iii) Hybridize Bio-Inspired Optimization with Lightweight Machine Learning for Cluster Management: Combining bio-inspired metaheuristics with streamlined machine learning techniques could achieve fast, efficient, and adaptive cluster formation and maintenance. Swarm intelligence or evolutionary algorithms can dynamically optimize cluster head selection and role rotation, while machine learning models tune parameters or predict traffic congestion and energy trends. This hybrid approach enables highly flexible, distributed coordination for clustering, while reducing computational burden, energy consumption, and time to convergence when compared to traditional heavyweight solutions [149].
(iv) Develop Multi-Objective, Explainable AI-Based Routing Frameworks and Promote Real-World Validation: Future research should pursue routing solutions that consider multiple, often conflicting, goals in real time. By designing explainable AI frameworks, networks can transparently weigh factors such as latency, load balance, energy use, and security, making the decision processes auditable and more reliable for operational deployment. Prototype routing solutions must be tested beyond simulation, in realistic and diverse field environments, to ensure models are robust, interpretable, and transferable from lab to field. The result will be trustworthy, versatile, and practically validated protocols for real IoT ecosystems [150].
(v) Edge Computing Integration: Integrating edge computing with WSN and IoT routing protocols opens new avenues for localized processing, aggregation, and decision-making. Allowing sensor nodes and clusters to perform computation, apply AI analytics, and adapt routing at the periphery can cut down core network congestion, reduce latency, and make real-time optimization feasible. Edge-enabled protocols also enhance privacy and data locality, enabling efficient use of distributed resources while scaling to support denser and more complex network applications [151]. This direction advances the vision of intelligent, decentralized, and self-managing IoT networks.

### 5.3. Emerging Trends

**Figure 8:**
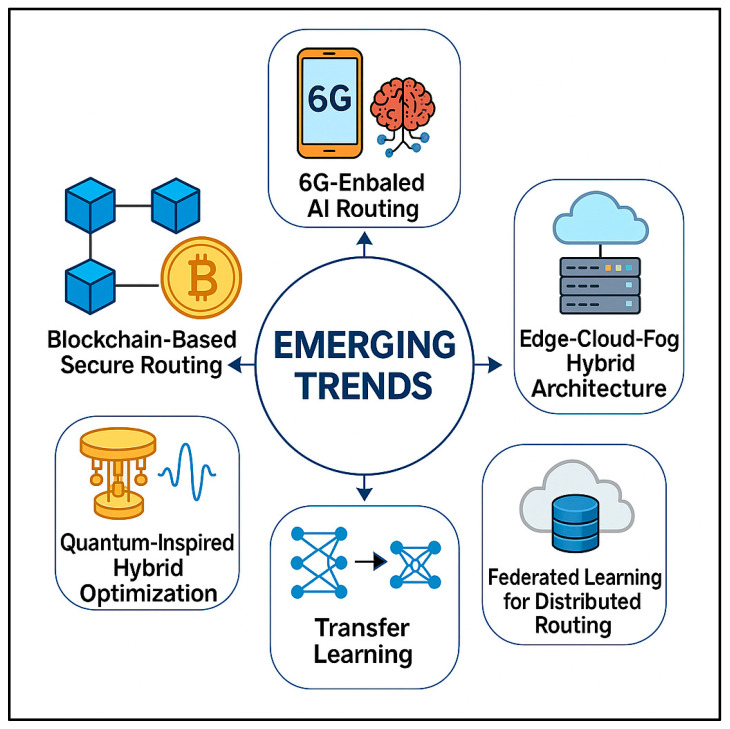
Recent emerging trends for routing in IoT-based WSN.

Emerging trends in energy-efficient routing for WSN-IoT networks are shaping the future of intelligent and adaptive communication systems. Figure 8 summarizes six key emerging trends that are being investigated to address the shortcomings of current WSN/IoT routing schemes. These include 6G-enabled AI routing, blockchain-based secure routing, transfer learning, quantum-inspired hybrid optimization, edge–cloud–fog hybrid architectures, and federated learning for distributed routing.

**Figure 9:**
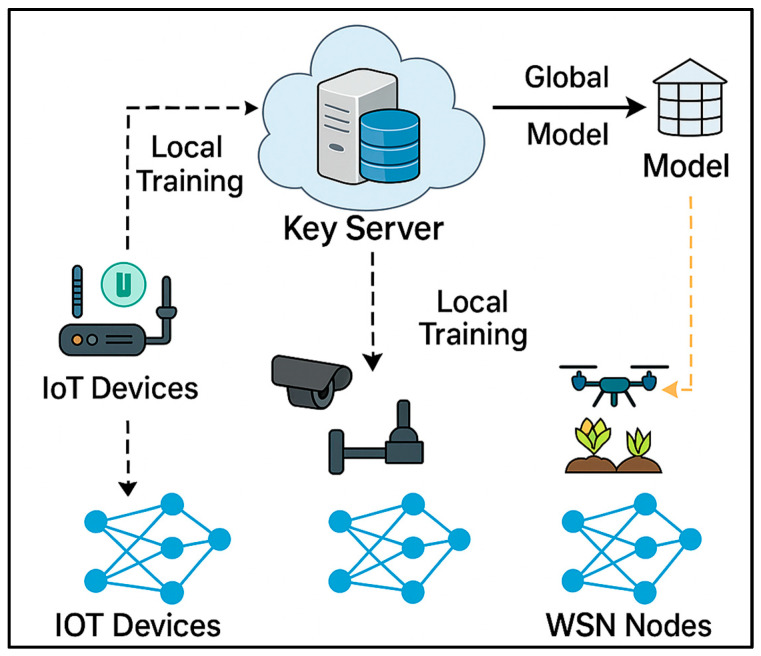
Architecture of Federated Learning in distributed routing.

(i) Blockchain-Based Secure Routing: Blockchain-based secure routing significantly enhances the security and trustworthiness of WSN by decentralizing and safeguarding routing information against threats such as unauthorized access, data tampering, and man-in-the-middle attacks [152]. Unlike traditional routing protocols that are often vulnerable due to their centralized nature and limited protection mechanisms, integrating blockchain enables each node or cluster head to securely record routing and cluster election data in an immutable ledger maintained by consensus among trusted nodes. This tamper-proof mechanism ensures that all network actions are transparently verifiable and resistant to manipulation, even in adversarial IoT environments. As a result, blockchain integration has been shown to notably improve both security and network longevity.
(ii) 6G-Enabled AI Routing: 6G-enabled AI routing represents a next-generation advance where the ultra-low latency, massive bandwidth, and intelligent orchestration features of 6G wireless networks synergize with advanced artificial intelligence to optimize WSN performance. In the context of IoT-based WSNs, 6G will provide real-time, high-reliability connectivity by leveraging native support for AI-driven protocols [153]. These technologies enable rapid network self-organization, context-aware decision-making, and robust security—even in highly dynamic and large-scale environments. AI-based routing, particularly modular and reinforcement learning frameworks, can adaptively balance energy consumption, minimize latency, and maximize packet delivery, addressing the challenges of resource-constrained nodes and ever-changing traffic demands. As such, 6G-enabled AI routing emerges as a critical trend for building future-proof, highly efficient, and resilient IoT sensor networks.
(iii) Edge-Cloud-Fog Hybrid Architecture: In Edge-Cloud-Fog hybrid architecture computation, storage, and analytics tasks are distributed dynamically across edge devices (sensor nodes), fog nodes (local micro-servers or gateways), and centralized cloud servers [154]. This multi-layer structure enables real-time data processing to close to the data source, drastically reducing latency as fog and edge nodes can respond instantly; studies report up to 40% faster response times for time-sensitive IoT applications compared to cloud-only designs. Offloading computation to edge and fog also conserves energy on resource-constrained sensors by minimizing long-distance transmissions: fog nodes can manage local data aggregation, predictive analytics, and even machine learning, which has been shown to improve energy consumption by up to 2–3 times in industrial deployments. Another benefit can be high scalability, as fog/edge nodes bridge massive numbers of heterogeneous sensors while maintaining flexible interoperability across protocols like ZigBee, Wi-Fi, Bluetooth, and cellular. As demand grows for mobility support, real-time responsiveness, and robust energy management in next-generation IoT networks, edge-cloud-fog architectures will become central to achieving both high performance and efficient, scalable sensor deployments.
(iv) Federated Learning for Distributed Routing: Federated Learning (FL) has emerged as a transformative approach enabling collaborative model training across distributed sensor nodes without centralizing sensitive data. Each node trains locally using its own observations, transmitting only learned model parameters (~0.4 KB) instead of raw data (~50 KB) to a central aggregator [155]. Figure 9 depicts a collaborative training paradigm where multiple IoT devices, drones, and sensor nodes locally train routing models on their own data without transmitting raw information to a central authority. A key server coordinates the process by aggregating locally trained model updates and computing a global model that is redistributed to all participants, enabling iterative refinement through repeated cycles of local training and global aggregation. This architecture reduces communication overhead by 60–70% and corresponding energy consumption by 15x while preserving privacy. Federated averaging algorithms aggregate local models into an improved global model broadcast back to all nodes. Despite slower convergence than centralized approaches, FL provides 2–5% quality degradation in exchange for massive energy and privacy benefits, making it ideal for large-scale, bandwidth-limited IoT deployments. Current research focuses on handling heterogeneous network conditions and reducing synchronization overhead for widespread adoption anticipated by 2025–2026.
(v) Quantum-Inspired Hybrid Optimization: Quantum-inspired hybrid optimization introduces principles from quantum computing such as superposition, entanglement, and advanced quantum search algorithms into classical metaheuristic optimization techniques for wireless sensor networks (WSNs) [156]. By integrating quantum-inspired algorithms like Quantum Genetic Algorithm (QGA) or Quantum Particle Swarm Optimization (QPSO), these approaches enable more efficient and balanced cluster head selection, proactive energy balancing, and faster convergence than traditional methods. Quantum-inspired techniques mostly use probabilistic search spaces and innovative initialization (e.g., Sobol sequences, Lévy flights) to better avoid local optima and promote uniform energy distribution, addressing persistent challenges such as energy hole prevention and dynamic node deployment. These advances offer scalable solutions for increasingly complex, large-scale IoT networks and pave the way for future integration with more advanced quantum computing hardware.
(vi) Transfer Learning: Transfer learning utilize knowledge or models from one domain, environment, or task and apply them to different but related scenarios without having to retrain from scratch [157]. This is particularly valuable for IoT-based WSNs, where training data can be limited, environments may shift rapidly, and computational resources are scarce. By reusing pretrained models or features from source tasks, transfer learning dramatically speeds up adaptation when new sensor nodes are deployed or when the network faces changing conditions. This makes transfer learning ideal for large-scale distributed networks, especially those with heterogeneous hardware or scenarios that require rapid adaptation, like industrial monitoring or smart agriculture. Importantly, transfer learning also enables more robust operation against concept drift (e.g., sensor aging or failure), supports specialized learning for diverse sensor types, and reduces the need for labeled data, addressing some of the most persistent challenges in practical WSN deployments.

## 6. Conclusions

This review paper highlights a detailed discussion of the existing routing algorithms from classic to Artificial Intelligence approaches and shows a critical role in IoT-based WSN applications and the growing need for energy-efficient (EE) protocols to enhance network lifespan and throughput. By evaluating both traditional protocols and emerging AI-driven strategies, the survey highlights how static routing methods fall short in dynamic environments and how intelligent optimization methods include metaheuristics, deep reinforcement learning, and bio-inspired clustering which tackle scalability, reliability, and energy constraints. Key gaps persist, notably in adaptive cluster management, multi-objective optimization, link quality prediction and robust security integration, especially for large-scale, heterogeneous deployments. Addressing these limitations with hybrid and context-aware solutions, supported by rigorous real-world validation, will be essential for the practical advancement of next-generation IoT sensor networks. This synthesis provides a foundation for future protocol design, encouraging further research into resilient, energy-conscious routing frameworks able to meet the evolving demands of diverse applications.

## Figures and Tables

**Table 1:** Classification of existing literature reviews on energy efficient routing optimization techniques.

Ref.	Type of Energy-Efficient Route Optimization Techniques					Clustering	AI-Driven Optimization	Application Area	Review Focused on
	NS	CL	MH	ML	DL				
[16]	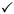	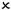	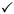	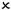	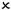	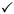	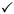	WSN routing	Comprehensive survey covering optimization strategies in routing, including trade-offs among cost, energy, and delay
[17]	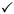	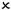	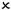	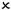	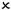	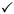	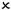	WSN	Overview of different routing schemes and their performance in WSN, covering latency, scalability, and energy use trade-offs.
[18]	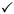	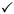	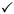	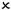	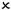	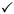	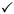	IoT applications	Comprehensive review of energy-aware IoT routing protocols with a focus on efficiency, protocol types, performance, and research gaps
[19]	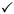	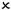	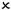	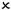	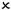	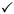	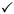	WSN-IoT	Detailed exploration of optimal CH selection techniques for enhanced energy efficiency.
[20]	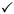	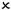	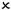	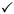	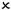	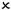	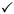	WSN-IoT	Application of Machine Learning in Localization for WSN-Assisted IoT with a Focus on Agricultural Monitoring.
[21]	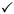	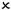	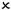	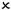	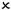	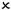	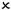	WSN	Comprehensive review of security threats and countermeasures in WSN routing, highlighting optimized secure communication.
[22]	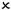	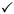	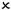	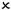	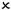	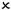	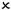	IoT applications	Comprehensive cross-layer review focusing on secure and low-latency communication methods across access, network, and application layers.
[23]	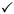	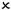	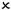	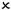	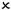	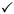	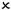	MANETs	Detailed analysis of load balancing in energy-sensitive multipath routing protocols.
[24]	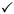	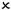	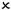	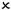	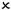	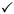	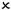	WSN	In-depth study of hierarchical routing protocols, focusing on energy conservation and extending network lifetime.
[25]	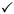	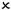	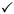	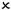	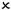	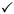	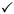	Energy- efficient WSN	Comprehensive review of bio-inspired hybrids for enhancing energy efficiency and prolonging lifetime in Wireless Sensor Networks (WSNs).
[26]	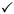	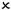	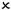	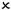	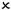	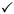	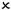	IoT systems	Survey covering various WSN techniques, including routing, energy efficiency, and network scalability.
[27]	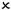	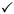	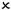	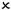	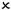	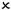	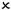	Next-gen IoT networks	Survey focusing on cross-layer secure communication with latency minimization in IoT.
[28]	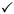	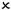	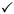	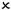	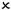	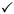	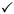	WSN-IoT	Study on Protocol-Level Energy Optimization in Large-Scale Networks.
[29]	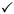	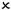	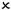	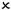	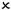	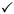	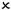	Query-driven WSNs	Exhaustive review of energy-efficient routing protocols employed in query-based Wireless Sensor Networks (WSNs).
[30]	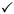	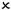	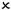	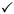	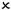	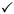	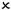	WSN-IoT	Bibliometric review using Web of Science dataset; maps publication trends, routing techniques, clustering and ML integration; compares protocols and identifies research trends.
[31]	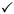	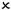	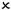	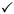	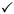	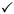	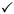		Review of ML and DL techniques for advanced WSNs; emphasizes DL development and applications
[32]	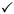	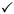	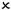	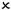	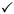	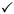	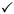		Focused on Reinforcement Learning specifically for WSN routing; energy efficiency through RL-driven decisions
[33]	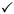	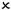	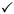	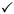	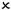	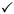	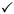		Systematic review of traditional, hybrid, and emerging optimization techniques
[34]	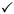	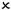	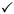	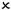	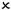	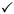	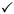		ML-based routing protocols specifically for WSN lifetime maximization; benefits, limitations, and network parameters
[35]	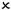	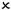	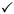	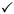	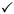	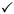	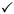		Energy-efficient clustering strategies uniquely integrating metaheuristics, ML, and DL
Our Work		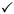		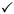	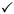	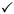	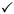	IoT-based WSN	This survey offers a comprehensive review of routing techniques in IoT-based WSNs, encompassing network structure, cross-layer design, meta-heuristics, machine learning, and deep learning. It also highlights existing challenges in WSN-IoT routing and outlines future research opportunities and potential solutions.

NS: Network Structure; CL: Cross Layer; MH: Metaheuristic; ML: Machine Learning; DL: Deep Learning.

**Table 2:** Communication layers and their roles in IoT-enabled WSN architecture.

Communication Level	Role	Routing Involved	Typical Communication	Technologies Used	Data Operations
Perception Layer	Sensing physical environment using sensor nodes	No	Sensor-to-gateway, Device-to-device (ZigBee, BLE, LoRa)	Sensors, RFID, Bluetooth, ZigBee, LoRa	Data collection and digital signal conversion
Middleware Layer	Data aggregation, protocol translation, and cloud interfacing	Minimal/ No	Gateway-to-cloud or Base station-to-server (IP-based protocols)	MQTT, CoAP, HTTP, Cloud services	Data filtering, coaching, load balancing, semantic processing
Network Layer	Routing and transmitting data across nodes to a sink/base station	Yes	Node-to-node, Cluster-to-sink (multi-hop routing protocols)	Routing protocols (LEACH, AODV, RPL), Wireless standards (802.15.4)	Path selection, energy-efficient forwarding, QoS maintenance
Application Layer	Presenting data to users or external systems through applications	No	User interface, API communication, cloud-to-app	Web/Mobile applications, Dashboards, REST APIs	Visualization, user notifications, and command actuation

**Table 3:** Comparison of traditional routing approaches and parameters.

Ref.	Network Structure	Cross-Layer Optimization	Cluster Head Selection	Energy Efficient	Multi-hop Routing	Mobility Support	Scalability	Research Gaps
[72]	✓	✗	✓	✓	✗	✗	✗	Does not resolve issues such as cluster head failure, scalability in large WSNs, inefficiencies in random cluster creation, security vulnerabilities, and adaptation to dynamic network conditions.
[73]	✓	✗	✓	✓	✗	✗	✗	Leaves gaps in real-time adaptivity, context-awareness, efficient data aggregation, and secure routing in highly mobile or variable IoT environments.
[74]	✓	✗	✓	✓	✗	✗	✗	Does not address advanced machine learning integration, security enhancement, or cluster-head election reliability under dynamic loads.
[75]	✓	✗	✓	✓	✗	✗	✗	Lacks mechanisms for security, handling intense mobility, and robust cross-layer integration needed for IoT/cloud deployments at scale.
[76]	✓	✗	✓	✓	✗	✗	✗	it does not fully address security, mobility, or energy balancing for nodes experiencing uneven traffic loads.
[79]	✓	✗	✓	✓	✗	✗	✗	Limited attention to energy consumption minimization and security integration in real-world IoT deployments.
[80]	✓	✗	✓	✓	✗	✗	✗	Needs further study in terms of energy efficiency, privacy, and data integrity under city-scale stress tests.
[81]	✓	✗	✓	✓	✗	✗	✗	Gaps remain in generic applicability, integration of security features, and adaptation for unpredictable event patterns.
[82]	✓	✗	✓	✓	✗	✗	✗	Lacks scalability, validation, and built-in adaptive defenses against network attacks.
[83]	✓	✗	✓	✓	✗	✗	✗	research gaps persist in end-to-end security, practical real-time event responsiveness, and field deployment studies.
[84]	✓	✗	✓	✓	✗	✗	✗	Leaves open challenges in lightweight cryptography, scalability, intra-cluster attacks, and context-aware adaptation.
[85]	✓	✗	✓	✓	✗	✗	✗	Fails to integrate cross-layer optimizations and dynamic mobility handling for non-uniform event patterns.
[86]	✓	✗	✓	✓	✗	✗	✗	Research still lacks real-world scalability tests, robust security features, and integration of AI for dynamic event response.
[87]	✓	✗	✓	✓	✗	✗	✗	Gaps persist in achieving seamless energy balance during rapid node movements, secure data aggregation, and adaptive hierarchical architectures.
[88]	✓	✗	✓	✓	✗	✗	✗	Real-world adaptability, collaborative energy scheduling, and robust, lightweight security are still underdeveloped.
[89]	✓	✗	✓	✓	✗	✗	✗	Leaves gaps in fine-grained energy management, privacy engineering, and validation for city-scale networks.
[90]	✓	✗	✓	✓	✗	✗	✗	Missing adaptive real-time mobility, next-gen security, and high-scale empirical deployment data.
[91]	✓	✗	✓	✓	✗	✗	✗	Lacks comprehensive multi-objective balancing and deployment across other verticals (limited scope)
[92]	✓	✗	✓	✓	✗	✗	✗	Fails to ensure lightweight, scalable privacy protocols are effective across diverse IoT hardware.

**Table 4:** Comparison of recent meta-heuristics CH-selection schemes in IoT-WSNs.

Ref.	Core Technique	Contribution	CH Selection Basis	Routing/Data Handling	Key Features	Limitations
[101]	Hybrid PSO + Cuckoo search	QoS-aware clustering with multipath routing	Energy + QoS fitness	Clustered, multipath	QoS supported; static sink	The data transmission process can be optimized using a swarm intelligence algorithm.
[102]	Compressive data fusion + clustering	Relay-assisted compressive fusion	Weighted unequal clustering	Multi-hop relay clustering	Energy saving; static sink	Relay nodes are selected based on energy and path loss. Optimization methods could enhance relay selection by energy, distance, and traffic.
[103]	Hybrid optimization for ICWSNs	Information-centric clustering with edge	Energy + distance	Clustered, edge-assisted	Edge-enabled; static sink	Six factors were considered for optimal CH selection. Coordination among them is crucial; multi-attribute approaches are required.
[104]	Fuzzy multi-criteria + bio-inspired routing	Adaptive fuzzy CH selection	Energy + distance + rank	Clustered, multi-hop	Robust clustering	The use of bio-inspired algorithms instead of Fuzzy rules may optimize the selection of CHs in a better way.
[105]	Optimized fuzzy clustering	Uncertainty-aware CH election	Energy + distance (fuzzy rules)	Clustered	Improved energy balance	The use of bio-inspired algorithms instead of Fuzzy rules may optimize the selection of CHs in a better way.
[106]	Improved Harmony Search	Throughput-optimized clustering	Energy + distance	Multi-hop clustered	Throughput focus	The network can be clustered to improve energy efficiency further.
[107]	Hybrid fault-tolerant multipath	Fault-resilient multipath routing	Energy + reliability	Clustered, multipath	Fault tolerance	Using deep neural networks is resource-consuming, leading to computational complexity and overhead on resource-constrained sensors.
[108]	Emperor penguin optimization + enhanced flower pollination	Joint fault diagnosis + CH routing	Energy + behavior indicators	Clustered, multipath	Fault detection	CHs with high energy usage form multiple routes, potentially increasing CH energy consumption
[109]	Neuro-fuzzy routing	QoS-aware clustering	Learned fuzzy rules	Clustered	QoS supported	Using neural networks may cause additional computational overhead on the resource-constrained sensors.
[110]	Hybrid GSA + CSA	Multi-sink optimization	Energy + delay	Clustered, multi-sink	Multi-sink supported	Swarm optimization guarantees better CH election than weightage-based fitness functions.
[111]	Hybrid ABC + DE	Load-balanced clustering for mobile sinks	Avg. energy + delay	Clustered	Mobile sink supported	Mobile sink movement needs location and clock synchronization, inducing routing overhead.
[112]	Enhanced ACO	Cluster + mobile sink path optimization	CH + pheromone reinforcement	Clustered, mobile sink	Latency optimized	CHs can be selected using swarm intelligence algorithms for better optimization.
[113]	GAPSO + SVM	IDSS-based clustering for IoT layer	Energy + location (SVM aided)	Clustered	Localization aided	Multi-hop communication can provide more energy efficiency.
[114]	Mobile agent-assisted clustering	Lifetime extension for heterogeneous WSNs	Heterogeneous energy tiers	Clustered, agent forwarding	Reliability focus	Mobile agents have bloating issues problem

**Table 5:** Comparative analysis of AI-driven energy-efficient routing techniques in IoT-enabled WSN.

Ref.	AI Technique	Energy Efficiency	Network Delay	Scalability	Link Prediction	WSN/IoT Environment	Limitations
[123]	Multi-Agent Reinforcement Learning (Q-learning)	✓	✓	✓	✓	Dynamic	High computational overhead, slower in large/mobile networks
[124]	Dynamic Objective Selection with RL (DOS-RL)	✓	✓	✓	✓	Dynamic	Frequent policy updates raise costs and scalability issues as networks grow
[125]	Tabu Search + ACO Hybrid	✓	✗	✓	✗	Static	Needs parameter tuning, cluster head depletion in topological changes
[126]	Swarm Intelligence (PSO, SI models)	✓	✓	✓	✗	Dynamic	Sensitive to initial values, synchronization overhead
[127]	Ant Colony Optimization (ACO Variant)	✓	✗	✓	✗	Dynamic	Multi-agent overhead, increased coordination required
[128]	Genetic Algorithm Optimization	✓	✓	✓	✗	Static	Iterative optimization slows for rapidly changing networks
[129]	Genetic Algorithm	✓	✗	✓	✗	Static	Slow adaptation, routing overhead in dynamic scenarios
[130]	Particle Swarm Optimization (PSO)	✓	✓	✓	✗	Dynamic	High computation needs, slow route updating
[131]	Quantum PSO + Fuzzy Logic	✓	✓	✓	✗	Dynamic	Increased complexity with combined fuzzy/quantum models
[132]	Neuro-fuzzy Data Routing (NFDR)	✓	✓	✗	✗	Dynamic	Degrades under rapid state changes
[133]	DRL + Graph Neural Network	✓	✓	✓	✓	Dynamic	High cost for training and operation
[134]	AI-based Modular Framework	✓	✓	✓	✓	Dynamic	Heavy overall demand for processing and data
[135]	CNN + BEA-SSA	✓	✓	✗	✗	Static	Security/complex routing increases delay
[136]	RNN (Path Planning/Optimization)	✓	✓	✓	✗	Dynamic	Not optimal for all dynamic topologies (e.g., moving sink)
[138]	Neural Network LEACH Variant	✓	✓	✓	✗	Static	Higher computational needs, heavier model
[139]	Greedy Discrete PSO (GMDPSO)	✓	✗	✓	✗	Dynamic	Adapts to mobiles, but slow when updating routes
[140]	Multi-Intel. Biomedical Routing	✓	✗	✗	✗	Static	Specific to biomedical routing; generalizability lacking
[137]	DRL with Graph Structure (GTD3-NET)	✓	✓	✓	✓	Dynamic	Resource-intensive, not yet validated in the field

**Table 6:** Quantitative performance benchmark of energy-efficient routing protocols in IoT-enabled WSNs.

Protocol (Ref.)	Routing	Energy/ Round (mJ)	Throughput (kbps)	Latency	PDR	Network Lifetime	Mobility
LEACH [72]	Traditional (Hierarchical)	85	45	120	92	1200	No
PEGASIS [73]	Traditional (Chain-based)	65	42	140	90	1800	No
NICC [81]	Cross-Layer	52	44	125	91	2100	Limited
PSO-LEACH [93]	Metaheuristic	55	46	110	94	2200	Limited
AO-FA Hybrid [97]	Metaheuristic (Hybrid)	48	50	95	97	3500	Yes
EER-RL [119]	Machine Learning (RL)	42	48	85	96	3200	Yes
Neuro-Fuzzy (NFDR) [132]	Deep Learning (Neuro-Fuzzy)	50	48	90	96	3000	Limited
CNN-BEA-SSA [135]	Deep Learning (CNN)	48	50	95	97	3500	No
RPP-RNN [136]	Deep Learning (RNN)	45	50	88	97	3300	Yes
DRL-GNN (TD3) [137]	Deep Learning (DRL+GNN)	35	52	72	98	4100	Yes

## Data Availability

The original contributions presented in this study are included in the article. Further inquiries can be directed at the corresponding author.

## Abbreviations

The following abbreviations are used in this manuscript:

ABC	Artificial Bee Colony
ACO	Ant Colony Optimization
AODV	Ad hoc On-demand Distance Vector
ASFO	Adaptive Swarm Firefly Optimization
BEA-SSA	Bald Eagle Assisted Sparrow Search Algorithm
BFO	Bacterial Foraging Optimization
CBRP	Cluster-Based Routing Protocol
CLEERDTS	Cross-Layer Energy-Efficient Reliable Data Transmission System
CL-IoT	Cross-layer Internet of Things Protocol
CNN	Convolutional Neural Network
CSA	Cuckoo Search Algorithm
CWSN-eSCPM	Cross-layer Wireless Sensor Network—Enhanced Service and Congestion Prediction Management
DL	Deep Learning
DRL	Deep Reinforcement Learning
DVRP	Distance Vector Routing Protocol
ECCM	Event-Cluster-based Cross-layer Management
EAR	Energy-Aware Routing
EER-RL	Energy-Efficient Routing with Reinforcement Learning
FDRL	Federated Deep Reinforcement Learning
FMCB-ER	Fuzzy Multi-Criteria Clustering and Bio-inspired Energy-Efficient Routing
GA	Genetic Algorithm
GAPSO	Genetic Algorithm and Particle Swarm Optimization
GEAR	Geographic and Energy Aware Routing
GNN	Graph Neural Network
GSA	Gravitational Search Algorithm
HEED	Hybrid Energy-Efficient Distributed
HSEERP	Hierarchical Secured Energy Efficient Routing Protocol
LEACH	Low-Energy Adaptive Clustering Hierarchy
LSP	Link State Protocol
MEC	Mobile Edge Computing
ML	Machine Learning
MH	Metaheuristics
MPNN	Message Passing Neural Network
NICC	Nature-Inspired Cross-layer Clustering
PEGASIS	Power-Efficient Gathering in Sensor Information System
PSO	Particle Swarm Optimization
QoS	Quality of Service
QPSO	Quantum Particle Swarm Optimization
REERP	Region-based Energy-Efficient Routing Protocol
RPL	Routing Protocol for Low Power and Lossy Networks
RPP-RNN	Rank-Based Path Planning with Recurrent Neural Network
RSSI	Received Signal Strength Indicator
SNN	Spiking Neural Network
SVM	Support Vector Machine
